# The role of caloric intake in the association of high salt intake with high blood pressure

**DOI:** 10.1038/s41598-021-95216-y

**Published:** 2021-08-04

**Authors:** Naftali Stern, Assaf Buch, Rebecca Goldsmith, Lesley Nitsan, Miri Margaliot, Ronit Endevelt, Yonit Marcus, Gabi Shefer, Itamar Grotto

**Affiliations:** 1 The Sagol Center for Epigenetics of Aging and Metabolism, Institute of Endocrinology, Metabolism and Hypertension, Tel Aviv-Sourasky Medical Center, Sackler Faculty of Medicine, Tel Aviv University, Tel-Aviv, Israel; 2 The Sackler Faculty of Medicine, Tel-Aviv University, Tel-Aviv, Israel; 3 Nutrition Department, Ministry of Health, Jerusalem, Israel; 4 Public Health Services, Ministry of Health, Jerusalem, Israel; 5 School of Public Health, University of Haifa, Haifa, Israel; 6 Faculty of Health Sciences, Ben-Gurion University of the Negev, Be’er Sheva, Israel

**Keywords:** Endocrinology, Risk factors

## Abstract

Since current recommendations call for a substantial reduction in overall sodium consumption, we tested whether or not these recommendations are implemented in common large subpopulations such as those with abnormal weight or hypertension in the current high sodium, high-calorie nutritional environment. In a national representative cross-sectional survey of the community-dwelling subjects aged 25–65 years conducted in Israel between 2015 and 2017, 582 randomly selected subjects completed health and dietary questionnaires, underwent blood pressure and anthropometric measurements and collected 24-h urine specimens, to assess dietary sodium intake. Overall mean 24-h sodium excretion was 3834 mg, more than double the recommended upper intake for adults < 1500 mg/day. Sodium excretion was directly related to caloric intake and blood pressure and linked to the presence of hypertension and overweight/obesity. The highest sodium excretion was seen in overweight/obese hypertensive subjects. This recent national survey shows a high consumption of sodium in the Israeli population and a dose–response association between caloric intake and urinary sodium excretion, independent of BMI and hypertension. Nevertheless, overweight/obese subjects with hypertension consume (excrete) more sodium than other BMI/ blood pressure-related phenotypes and may thus comprise a target subpopulation for future efforts to reduce sodium intake.

## Introduction

High sodium intake is associated with high blood pressure and an increased risk of cardiovascular disease and mortality^1,2^. A decrease in sodium intake has been shown to contribute significantly to blood pressure reduction in people with and without hypertension and to a reduction in the rate of stroke and cardiovascular mortality^3–5^. The American Heart Association (AHA) and the World Health Organization (WHO) recommended that daily sodium intake should be lowered to < 1500 and < 2000 mg/d, respectively^6,7^. Prior to the publication of these recommendations, the global mean sodium intake was estimated to be 3.95 g/day, twice or higher than the recommended levels^2^.

Of all methods used to asses salt intake^8^ in population surveys, 24-h urinary sodium excretion (24hNa) is undisputedly the best as apparently 90% of ingested sodium is excreted in the urine^9,10^. In terms of public health, it is critical to understand whether or not internationally set recommendations for sodium consumption are being gradually implemented. Two recent reports from Australia and the US, two major Western, Anglo-Saxon yet multiethnic societies, suggest that they are not: the mean sodium intake based upon 24hNa in these reports was about 3.6 g/d^11,12^. Further insight into the apparent persistent excessive sodium consumption could be gained by examination of the relation between sodium intake, nutrition and the presence of common phenotypes such as obesity and hypertension in adults in Western societies.

The Israel Ministry of Health commissioned a national sodium intake survey based on 24-h urinary sodium excretion, with a planned repeat survey in the future, to assess the impact of the sodium reduction program^13,14^. The aims of the survey which are addressed in the present report were to (a) determine sodium intake among Israeli adults, using 24hNa data as well as dietary evaluation, in a population exclusive of subjects using medications that can alter sodium excretion; (b) assess how sodium intake is related to blood pressure, hypertension, obesity and caloric intake.

## Methods

### Sample and population

The survey was carried out by the Nutrition Division and the Health Promotion Department of the Public Health Services of the Ministry of Health and the Institute of Endocrinology, Metabolism and Hypertension, Tel Aviv Sourasky Medical Center. A representative sample of Israeli adults of two age groups, 25–44 and 45–64 years, Jews and Arabs, comprised the basis of this report. The sample size was determined so that in a repeat survey it would be possible to identify a reduction in daily salt consumption from 9.6 g to approximately 6 g at the program conclusion, assuming that other interviewees would then be included. To obtain a power of at least 80% and to test the hypotheses at a significance level of 5%, a minimum of 140 people in each of the four groups (2 age groups, 2 sex groups) were sampled. The required sample size was 560, and the sample size was set to 630 people in anticipation of about 10% incomplete urine collections. The sample population comprised subjects insured in three major Health Maintenance Organizations (HMOs): Clalit Health Services, Maccabi Health Services and Leumit Health Services, which collectively insured 86.1% of the population^15^.

Interviewees were sampled from six out of the seven state districts in five localities per district, each with at least 20,000 residents. All randomly sampled subjects in the pre-determined age range and survey locations were included. Exclusion criteria were the use of diuretics, angiotensin-converting enzyme inhibitors, angiotensin receptor blockers or steroids; a known diagnosis of kidney failure, heart failure, stroke or liver disease; pregnant women, or those with urinary incontinence. The survey had approval of the Helsinki's (human subject) committee of the Tel Aviv-Sourasky Medical Center (approval number 0020-12-TLV), Ethics committee of the Clalit Health Services (approval number 0010-12-COM), Maccabi Health Services (approval number 2013025) and Leumit Health Care Services (approval no. 001-13). Written informed consent was obtained from all subjects participating in the survey. All methods were carried out in accordance with STROBE guidelines for cross-sectional studies^16^. All methods were performed in accordance with the relevant guidelines and regulations by the Declaration of Helsinki.

### Survey protocol

The lists of the sampled persons received from the HMOs were combined and letters sent with a brief explanation, shortly after which, interviewees were called, received an explanation regarding the 24-h urine collection and a monetary compensation ($100) for the collection. The first interview was set at the interviewee's home after a verbal consent to participate was given, during which participants signed the Informed Consent Form and were introduced to and provided with the required equipment for the 24-h urine collection along with a detailed explanation of the urine collection protocol (Supplemental Methods 1). The second meeting was on the day of completion of the 24-h urine collection. An hour-long interview included a general detail personal and health questionnaire, two dietary questionnaires and anthropometric measurements.

### Measures

***Anthropometric outcomes:*** Height and weight were measured using a digital scale and a measuring tape as previously described^17^. ***The general questionnaire*** included questions on salt usage, knowledge and attitudes regarding sodium andsalt, socio-demographic data, heath status, exercise habits, medications and smoking (Supplemental Methods 2).

***The nutrition questionnaires*** consisted of an interviewer-administered 105 items specially adapted food frequency questionnaires (FFQ), (Supplemental Methods 3) and a 24-h dietary recall of the day of the urine collection, i.e. the day prior to the interview (Supplemental Methods 2). The 24-h dietary recall questionnaires were then transferred to the Nutrition Division, Ministry of Health into the “Tzameret” dietary analysis program^18^ as were the FFQs and the general questionnaires.

***Blood pressure and heart rate*** were measured with an OMRON digital monitor (OMRON M2) by personnel trained by the Ministry of Health staff. Following 5 min in a stable seating position, two measurements were taken, and where the difference was greater than 10% for any of the measures (systolic/diastolic/pulse), a third measurement was carried out, and the average of the last two measurements calculated. For classification of blood pressure measurements during the survey, hypertension was defined as levels ≥ 130/80 mmHg (American College of Cardiology/ American Heart Association; ACC/AHA 2017 definition) or ≥ 140/90 mmHg (European Society of Hypertension, ESH, 2018 definition). Hypertension was also defined for parallel analyses by a known diagnosis of hypertension of which the interviewees had been aware and/or by the active use of anti-hypertensive medications.

***Urine samples:*** The total urine volume was recorded as detailed in the Supplemental Methods 4. Urine sodium concentration was measured and after the calculation of total sodium excreted, in mg, the obtained valued was divided by the ratio of the atomic weight of sodium [= 23] to the molecular weight of sodium chloride [= 58.45;"salt"], i.e., 0.393, as nearly all urinary sodium is excreted as sodium chloride.

### Statistical analyses

We used the SPSS program, Version 25. Frequency distributions were calculated, and continuous variables presented as mean ± SD and, in certain cases, followed by median and inter quartile range. Categorical variables are presented as percentages. Chi square test was applied for categorical variables and T-test or One-way analysis of variance (ANOVA) for continuous variables (compared between dichotomous or categorical groups). The association between sodium excretion and both measured and diagnosed hypertension was analyzed by (a) univariate association testing the mean levels of systolic and diastolic blood pressure across sodium excretion categories; (b) stratification to BMI and hypertension groups and analysis of the calorie-normalized sodium excretion; (3) multivariate approach with the implementation of both linear and logistic regressions predicting blood pressure levels or hypertension as a function of sodium excretion or sodium excretion levels as function of caloric intake and other variables. To minimize information/report bias when kcals were included in certain analyses, subjects whose caloric intake was less than 800 kcals/day (n = 23) or whose BMI was < 16 kg/m^2^ (n = 17) were considered as outliers or reflecting measuring/data entry errors and were thus excluded. Since missing data on outcomes was < 6%, chance of bias related to incomplete data was low (Supplemental Methods 5).

### Ethics approval and consent to participate

The survey had Ethics committees' approvals from each of the HMOs involved in the survey. Informed consent was obtained and countersigned by the interviewer.

## Results

### Characteristics of the patients

Of the initial sample of 3985 individuals, 582 had valid 24-h urine collections (Figure S1). Due to problems in locating participants only 6.3% of the participants were Arabs compared to 14.2% for the same age groups in the population. The sex distribution in the sample was similar to the distribution in the population (men 43.8%, women 56.2%). The results listed in Tables 1 and 2 are for those with valid urine collections.

**Table 1:** The socio-demographic and physical characteristics of the sample with urine tests.

		All (N = 582)	Men (N = 266)	Women (N = 316)
Age (years)	Mean (SD)	46.18 (12.15)	46.26 (12.89)	46.10 (11.5)
Sex	%	–	45.78	54.2
Ethnicity	Jewish; n (%)	549	242 (91)	307 (97.2)
	Other; n (%)	33	24 (9.0)	9 (2.8)
Education (years) (n = 577)	Mean (SD), IQR	14.7 (3.2), 5–32	14.6 (3.2), 8–32	14.9 (3.13), 5–26
Education	No certificate; n (%)	60 (10.3)	31 (11.7)	29 (9.2)
	Matriculation; n (%)	187 (32. 1)	78 (29.3)	109 (34.5)
	Diploma; n (%)	58 (10.0)	36 (13.5)	22 (7.0)
	Academic Degree; n (%)	274 (47.1)	121 (46.5)	153 (48.4)
Marital status	Married/living with a partner; n (%)	430 (73.9)	198 (74.4)	232 (73.4)
Religiosity	Secular; n (%)	308 (52.9)	139 (52.3)	169 (53.5)
	Traditional; n (%)	158 (26.8)	74 (27.8)	82 (25. 9)
	Orthodox; n (%)	77 (13.2)	38 (14.3)	39 (12.3)
	Ultraorthodox; n (%)	24 (4.1)	8 (3.0)	16 (5 .1)
	Other; n (%)	17 (2.9)	7 (2.6)	10 (3.2)
Socio-economic status (personal monthly income), in NIS ($1 = 3.5 NIS, approx.)	Low (< = 6000); n (%)	145 (24.9)	42 (15.8)	103 (32.6)
	Middle (6001–10,000); n (%)	173 (29.7)	81 (30.5)	92 (29.1%)
	High (more than 10,000); n (%)	127 (21.8)	82 (30.8)	45 (14.2)
	Not relevant/refuse; n (%)	137 (23. 5)	61 (22.9)	76 (24.1)
Height	cm; Mean (SD), IQR	167.9 (9.7), 108–196	174.7 (7.3), 147–196	162.3 (7.7), 108–188
Weight	Kg; Mean (SD), IQR	75. 8 (16.7), 33.5–151.4	83.0 (15.3), 50.7–147	69.6 (15.4), 33.5–151.4
BMI	kg/m^2^; Mean (SD), IQR	26.78 (5.1), 13.8–52.4	27.17 (4.5), 16.8–49.4	26.45 (5.5), 13.8–52.4
Blood pressure (measured)	Systolic; Mean (SD), IQR	124.4 (18.6), 84–195	129.8 (17.2), 99–195	119.9 (17.8), 84–180
	Diastolic; Mean (SD), IQ R	80.5 (11.4), 48–135	83.8 (11.4), 58–128	77.8 (10.7), 48–135
Smoking status	Current; n (%)	134 (23.0)	67 (25.2)	37 (21.2)
Physical activity	Meet recommendation*; n (%)	155 (26.6)	78 (29.3)	77 (24.4)
Health status (ever diagnosed)- based on self-report	Diabetes; n (%)	65 (11.2)	35 (13.2)	30 (9.5)
	Coronary heart disease; n (%)	19 (3.3)	14 (5.3)	5 (1.6)
	Stroke; n (%)	23 (2.2)	9 (3.4)	4 (1.3)
	Hypertension; n (%)	89 (15.3)	50 (18.8)	39 (12.3)

*BMI* Body Mass Index, *IQR* Inter Quartile Range, *NIS* New Israeli Shekels, *SD* Standard Deviation.

*As recommended by the American College of Sports Medicine (ACSM)-150 weekly minutes or more of moderate intensity or 75 weekly minutes of intense physical activity.

**Table 2:** Sodium excretion (mg) by demographic and health characteristics—as measured in the 24-h urine collection.

		Mean (SD)	IQR	Median	Pvalue
All	(n =582)	3834 (1690)	2645–4755	3542	–
Sex	Male (n = 266)	4374 (1844)	3059–5405	4094	P < 0.001 (men vs. women)
	Female (n =316)	3380 (1397)	2323–4180	3197	
Age (years)	≤ 34 (n = 127)	3623 (1536)	2392–4554	3519	n/s between groups
	35–44 (n = 139)	3937 (2001)	2484–5083	3404	
	45–54 (n = 153)	3974 (1647)	2932–4968	3588	
	≥ 55 (n = 163)	3780 (1543)	2668–4646	3634	
Socio-economic status*	Low (n = 145)	3467 (1558)	2173–4404	3243	^p < 0.01, between the low and high SES groups
	Middle (n = 173)	4147 (1764)	2829–5175	3864	
	High (n = 127)	4018 (1666)	2898–5014	3772	
	Refused/not relevant (n = 137)	3657 (1669)	2518–4266	3358	
BMI (kg/m^2^)	Normal weight (18.5–24.99; n = 211)	3527 (1610)	2346–4358	3220	P = 0.001 between normal and obese groups
	Overweight (25–29.99; n = 213)	3834 (1725)	2691–4519	3496	
	Obese (30–34.99; n = 92)	4380 (1720)	3157–5296	4094	
	Morbid Obese (= 35; n = 33)	4304 (1337)	3162–5140	4416	
Caloric Intake (Kcals/day)^†^	First quartile (812—1275; n = 123)	3176 (1292)	2070–4025	3151	P < 0.0001 between Q1 and Q3 and between Q1 and Q4
	Second quartile (1277—1747; n = 146)	3739 (1489)	2622–4502	3438	
	Third quartile (1751—2324; n = 146)	4139 (1853)	2823–5048	3726	
	Fourth quartile (2333—6234; n = 145)	4269 (1689)	3013–5186	3956	
Intake- by meeting physical activity recommendations	Yes (n = 155)	3849 (1710)	2714–4738	3542	n/s between groups
	No (n = 427)	3829 (1684)	2599–4807	3542	
Hypertension (ever diagnosed)- based on self-report	Yes (n = 89)	3968 (1707)	2783–4772	3703	P = 0.4265
	No (n = 492)	3812 (1691)	2628–4790	3507	
Measured elevated BP	Normal (n = 406)	3703 (1699)	2478–4600	3358	P = 0.003
	Elevated (≥ 140/90 mm/Hg; n = 143)	4180 (1583)	3128–5175	3864	
Taking BP medications (self-reported)^‡^ (n = 89)	Yes (n = 46)	4080 (1849)	2507–4864	3760	n/s
	No (n = 43)	3841 (1529)	2806–4830	3588	
Diabetes (ever diagnosed)- based on self-report	Yes (n = 64)	4081 (1774)	3001–4956	3611	P = 0.216
	No (n = 516)	3812 (1691)	2622–4732	3542	
Coronary heart disease (ever diagnosed)- based on self-report	Yes (n = 19)	3932 (1706)	2668–6072	3243	P = 0.808
	No (n = 562)	3836 (1690)	2645–4738	3542	

*BMI* Body Mass Index, *BP* Blood Pressure, *IQR* Inter Quartile Range, *SD* Standard Deviation.

*As indicated in Table 1.

^†^Excluding individuals with caloric intake < 800 kcals.

^‡^Of those reporting having hypertension.

### Outcomes

#### 24 h Sodium excretion: a general overview

The overall mean 24hNa was 3834 mg (Table 2), which reflects ~ 9.76 g of salt (sodium chloride), with a median excretion of 3542 mg. Sodium excretion was related to sex, BMI, caloric intake, blood pressure, the presence of hypertension and the socio-economic status, but not to the age of the surveyed population (25–65 years).

##### Sex

Sodium excretion was higher in men than in women (4374 vs. 3380 mg; p < 0.001) (Table 2), and this difference persisted after adjustment for kg body weight (p < 0.05), or creatinine levels (3340 vs. 2934 mg per gram creatinine/24 hours; p < 0.001), but not after adjustment for caloric intake (excretion per 1000 cal consumed) (2384 vs. 2307 mg per 1000 Kcals, p = 0.538).

##### Caloric intake

**Figure 1:**
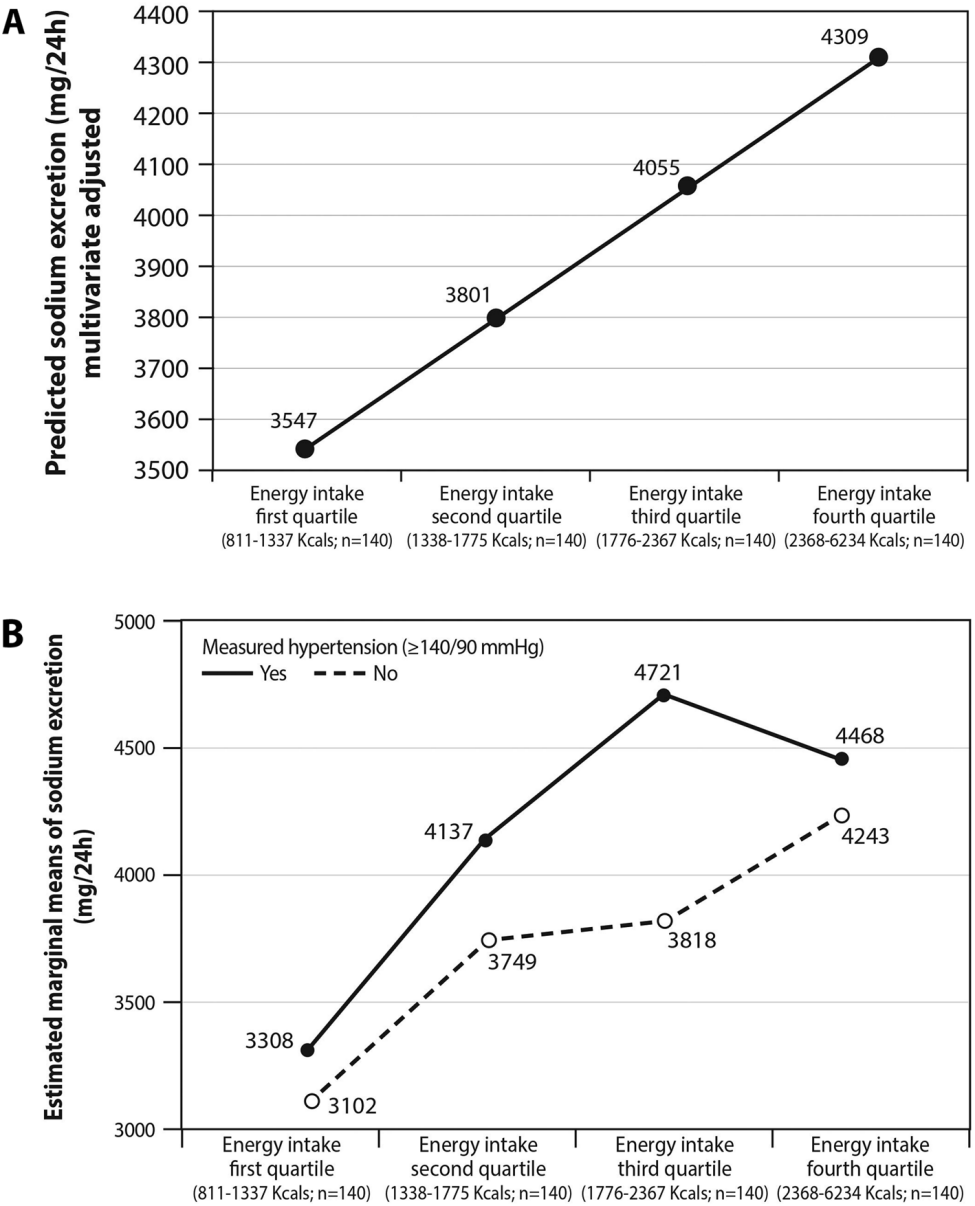
Sodium excretion as a function of energy intake, adjusted for other explanatory variables (**A**); The interaction between energy intake and the presence of hypertension in relation to sodium excretion—a two-way ANOVA analysis (**B**). (**A**) The line shows the predicted values of sodium excretion in the different energy intake quartiles, adjusted to other explanatory/confounding variables which can be seen in the formula below. Values represent the scenario where each coefficient of the explanatory variables is multiplied either by "1" or "0". Variables with coefficients multiplied by "1" were energy quartile and BMI. The rest were multiplied by "0". Multivariate linear regression formula: $Y=2698\,+\,254\,*\,\left(energy \,quartile\right)\,+\,145\,*\,\left(Measured \,hypertension \,\ge\, 140/90\,=\,1 \,vs. \,other\,=\,0\right)\,+\,595\,*\,\left(BMI\ge 30\,=\,1 \,vs. \,<\,30\,=\,0\right)\,+\,665\,*\,\left(sex, \,male\,=\,1;\,female\,=\,0\right)\,-\,410\,*\,\left(CHD \,diagnosis., \,yes\,=\,1;\,no\,=\,0\right)\,+\,256\,*\,\left(Diabetes \,diagnosis , \,yes\,=\,1;\,no\,=\,0\right)\,-\,0.318\,*\,\left(age,\,\ge\, 45\, =\,1;\,else\,=\,0\right)\,+\,95\,*\,(ideal \,physical \,activity \,by \,ASCM \,recommendations, \,yes\,=\,1;\,no\,=\,0).$ Energy intake was positively linked to increase in sodium excretion (p < 0.001, R^2^ = 13.1%). (**B**) Energy intake was positively associated with an increase in sodium excretion regardless of hypertension status (P < 0.001 across quartiles). Hypertension (≥ 140/90 mmHg) was also positively linked to sodium excretion (p = 0.006 between hypertensive compared to normotensive subjects). Indicated by the relatively parallel lines, as well as by Between-Subjects Effects Test, there was no overall interaction between caloric intake and hypertension in relation to sodium excretion (p = 0.361) suggesting that the joint effect of hypertension and caloric intake the combined is less than the sum of their individual effects (no synergistic effect on sodium excretion). Error variance of the sodium excretion is equal across groups using the Levene's Test for homogeneity (p = 0.064).

Figure 1A shows a linear relationship between caloric intake (in quartiles) and sodium excretion as the dependent variable, after adjustment for sex, BMI, the presence of hypertension, diabetes and coronary heart disease (CHD), age and physical activity (p < 0.001; R = 36.2%; R^2^ = 13.1%). Although bread, vegetables (cooked included), cheese and poultry were the main sources of sodium (Figure S2), they still accounted for less than half of the consumed salt. Energy intake was positively associated with an increase in sodium excretion in those without or with hypertension (P < 0.001 across quartiles). Indicated by the relatively parallel lines, as well as by Between-Subjects Effects Test, there was no overall interaction between caloric intake and hypertension in relation to sodium excretion (p = 0.361): the combined effect of hypertension and caloric intake is less than the sum of their individual effects (Fig. 1B). No interaction was detected for BMI and caloric intake in relation to sodium excretion (Figure S3).

The association of sodium excretion with caloric intake was also examined by multivariable linear regression model with sodium excretion (mg/day) as a dependent variable and multiple variables as covariates (Table 3). In this analysis, sodium excretion could be predicted by several covariates: caloric intake, BMI and sex (male vs. female) were each independently and positively associated with an increase in sodium excretion. Independent ofall other covariates in the model and extrapolated by the findings of the linear regression within the range of caloric intake in the sample (812–6234 kcals), for every 100 additional Kcal consumed, sodium excretion increased by 28.6 mg/day of sodium (Table 3, model 4).

**Table 3:** Prediction of sodium excretion by blood pressure levels (systolic and diastolic), BMI and energy intake adjusted for other explanatory variables: a multivariate linear regression.

	Unstandardized Coefficient	95% CI for coefficient^†^
**Model 1**		
Systolic blood pressure (mm/Hg)	7.929	− 2.611–18.468
Diastolic blood pressure (mm/Hg)	14.560	− 2.331–31.452
**Model 2**		
Systolic blood pressure (mm/Hg)	3.139	− 7.581–13.858
Diastolic blood pressure (mm/Hg)	12.247	− 4.487–28.980
*BMI (kg/m*^*2*^*)*	*56.863*	*26.651–87.05*
**Model 3**		
Systolic blood pressure (mm/Hg)	2.960	− 7.532–13.453
Diastolic blood pressure (mm/Hg)	10.563	− 5.830–26.956
*BMI (kg/m*^*2*^*)*	*52.588*	*22.967–82.210*
*Energy (Kcals)*	*0.422*	*0.252–0.593*
**Model 4**		
Systolic blood pressure (mm/Hg)	1.462	− 9.483–12.407
Diastolic blood pressure (mm/Hg)	5.644	− 10.683–21.971
*BMI (kg/m*^*2*^*)*	*59.517*	*29.593–89.441*
*Energy (Kcals)*	*0.286*	*0.108–0.463*
Age (years)	− 6.243	− 18.626–6.141
*Sex (male = 1; female = 0)*	*641.767*	*381.76–656.11*
coronary heart disease (yes = 1; 0 = no)	− 363.695	− 1221.247–493.857
Diabetes (yes = 1; 0 = no)	186.232	− 283.112–655.576
Perform ideal physical activity*	150.449	− 162.525–463.424

Multivariable linear regression model with sodium excretion (mg/day) as a dependent variable and multiple variables as covariates using Enter method with 4 blocks: first block included blood pressure; second block included BMI; third block included energy intake and fourth block including age, sex, coronary heart disease, diabetes and physical activity.

The unstandardized coefficient represents the amount of change in sodium excretion (in mg/day) due to a change of 1 unit of covariate. For example, independent from all other covariates in the model, for every 1 additional Kcal sodium excretion was increased by 0.286 mg/day.

*As recommended by the American College of Sports Medicine (ACSM)-150 weekly minutes or more of moderate intensity or 75 weekly minutes of intense physical activity.

^†^Significance is indicated by CI wrriten in italics

*BMI* Body Mass Index, *CI* Confidence Interval.

#### Blood pressure and hypertension

Since the study design dictated the exclusion of subjects treated with drugs that potentially affect urinary sodium excretion such as diuretics, the rate of known hypertension (15.7%) underestimated the true prevalence of hypertension in this age group. Based on measurements of blood pressure during the survey, 26% of the interviewees were hypertensive, as defined by ESH (≥ 140/90 mmHg) and 54.5% hypertensive according to the AHA/ACC definition (≥ 130/80 mmHg).

**Figure 2:**
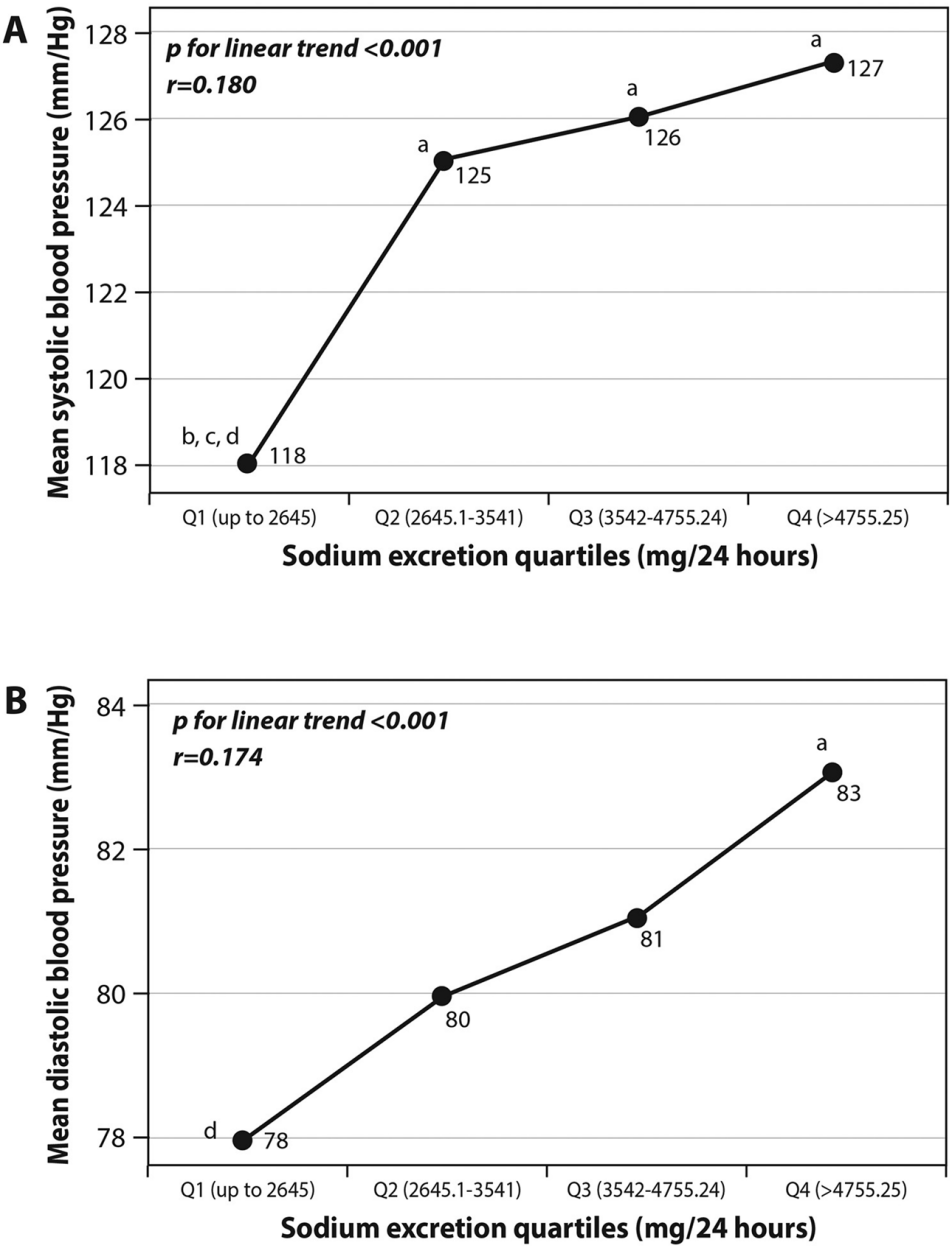
(**A**,**B**) univariate associations between sodium excretion quartiles and systolic (**A**) or diastolic (**B**) blood pressure. Statistical differences between sodium excretion levels (analyzed using One-Way Analysis of variance [ANOVA]) are represented by the following letters: a = significantly different than Q1 (p < 0.05); b = significantly different than Q2 (p < 0.05); c = significantly different than Q3 (p < 0.05); d = significantly different than Q4 (p < 0.05).

In univariate analysis (Fig. 2), systolic and diastolic pressures were related to the quartiles of sodium consumption (excretion rates). In a linear regression analysis predicting the increase of either systolic or diastolic blood pressure, there was an independent significant association between sodium excretion quartiles and both systolic and diastolic blood pressure, adjusted for diabetes, CHD, age, energy intake, smoking, physical activity and BMI (Table 4). For each increase in the sodium excretion quartile level, systolic and diastolic blood pressure increased by 1.324 (p = 0.045) and 0.978 (p = 0.025) mm/Hg, respectively.

**Table 4:** Prediction of measured blood pressure by sodium excretion levels (as quartiles or according to DRI upper-level recommendations) adjusted for other explanatory variables and stratified according to sex—results from a multivariate linear regression.

		All (N = 539)	Men (N = 247)	Women (N = 297)
Beta coefficients; 95% CI (p*)				
Sodium excretion levels (quartiles)**	Systolic BP	*1.324; 0.032, 2.617* *(p*** = ***0.045)*	0.220; − 1.707, 2.146 (p = 0.822)	0.680; − 1.044, 2.405 (p = 0.438)
	Diastolic BP	*0.978; 0.123, 1.834* *(p*** = ***0.025)*	0.870; − 0.416, 2.156 (p = 0.184)	0.231; − 0.924, 1.386 (p = 0.694)
Sodium excretion according to the DRI^†^	Systolic BP	*4.516; 1.006, 8.026* *(p*** = ***0.012)*	1.385; − 4.772, 7.542 (p = 0.658)	*4.480; 0.368, 8.593* *(p*** = ***0.033)*
	Diastolic BP	1.775; − 0.560, 4.109 (p = 0.136)	0.109; − 4.018, 4.236 (p = 0.958)	1.912; − 0.853, 4.676 (p = 0.175)

*Significant beta coefficient value is considered as p < 0.05 and wrriten in italics

**Q1 (up to 2645); Q2 (2645.1–3541); Q3 (3542–4755.24); Q4 (> 4755.25).

^†^Defined as 0 (below 2400 mg/day) or 1 (≥ 2400 mg/ day) – based on upper limit suggested by the DRI.

Models adjusted to: diabetes presence (0 = no; 1 = yes), coronary heart disease presence (0 = no; 1 = yes), age, energy intake, smoking (0 = no or in the past; 1 = yes), ideal physical activity (As recommended by the American College of Sports Medicine [ACSM]-150 weekly minutes or more of moderate intensity or 75 weekly minutes of intense physical activity; 0 = no; 1 = yes) and BMI categories (1. up to 18.5 2. 18.5–24.99 3. 25–29.99 4. 30–34.99 5. above or equal 35).

*BP* Blood Pressure, *CI* Confidence Interval, *DRI* Dietary Reference Intakes.

**Figure 3:**
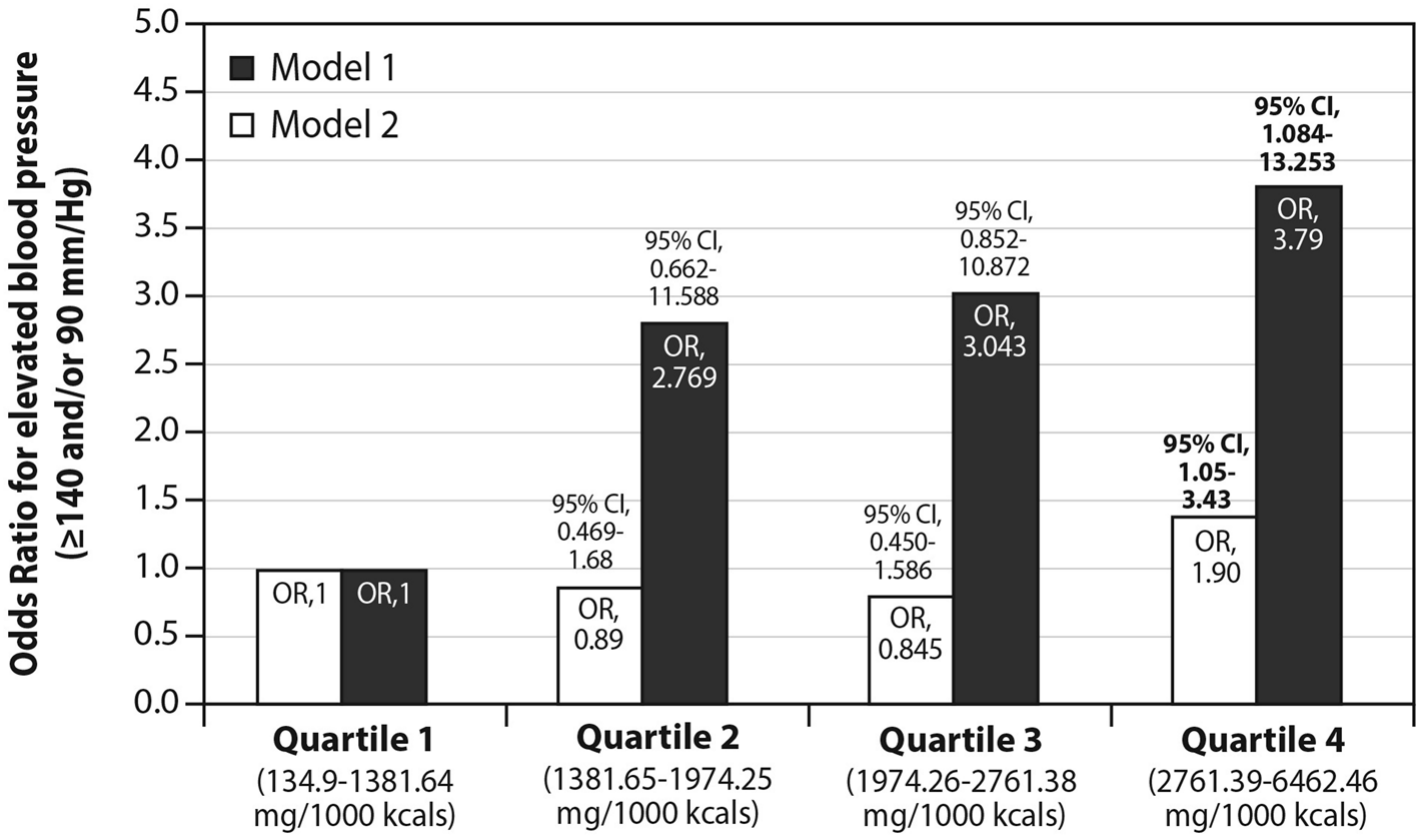
The independent association between sodium excretion (Model 1, solid bars) as well as sodium density (excretion per 1000 kcals; Model 2, open bars) and measured hypertension (≥ 140 and/or 90 mm/Hg). Results from a multivariate logistic regression indicating the higher odds for elevated blood pressure (≥ 140 and/or 90 mm/Hg) as a function of highest sodium excretion (per 1000 Kcals)—independent of other explanatory variables (age, sex, BMI categories, coronary heart disease, diabetes, ideal physical activity and smoking status). As compared to the lowest level (Q1), the highest level of sodium excretion (represented as Q4) was significantly and positively associated with higher odds for hypertension. Adjusted for: the presence of diabetes (0 = no; 1 = yes), coronary heart disease (0 = no; 1 = yes), age (up to 43.9 = 0; 44–65 = 1), smoking (0 = no or in the past; 1 = yes), ideal physical activity (As recommended by the American College of Sports Medicine [ACSM]-150 weekly minutes or more of moderate intensity or 75 weekly minutes of intense physical activity; 0 = no; 1 = yes) and BMI categories (1. up to 18.5 2. 18.5–24.99 3. 25–29.99 4. 30–34.99 5. above or equal 35 [category 1 = reference]) and sex (0 = female; 1 = male).

Compared to interviewees excreting sodium at the lowest quartile, subjects at the highest quartile of sodium excretion had nearly four folds odds (odds ratio [OR] = 3.79; 95% CI-1.08–13.25) for the presence of measured hypertension, but after normalization for caloric intake, i.e., sodium excreted per 1000 cal consumed [i.e., sodium density], the OR dropped to 1.9 (95% CI 1.05—3.42; p = 0.03). Hence, sodium density, not only overall sodium excretion, was linked to hypertension. These comparisons were made using a multivariate regression with adjustment for the presence of diabetes, CHD, age group, current smoking, physical activity, sex and BMI (Fig. 3). Finally, caloric intake alone was not related to either blood pressure (ß = 0.001; p = 0.103 for systolic and ß = 0.001; p = 0.124 for diastolic) or the presence of hypertension (OR for hypertension > 1 for each energy quartile compared to the lowest quartile, p > 0.1 for all), when adjusted for confounders (age, sex, BMI, sodium excretion, diabetes, CHD, physical activity and smoking).

#### BMI and obesity

**Figure 4:**
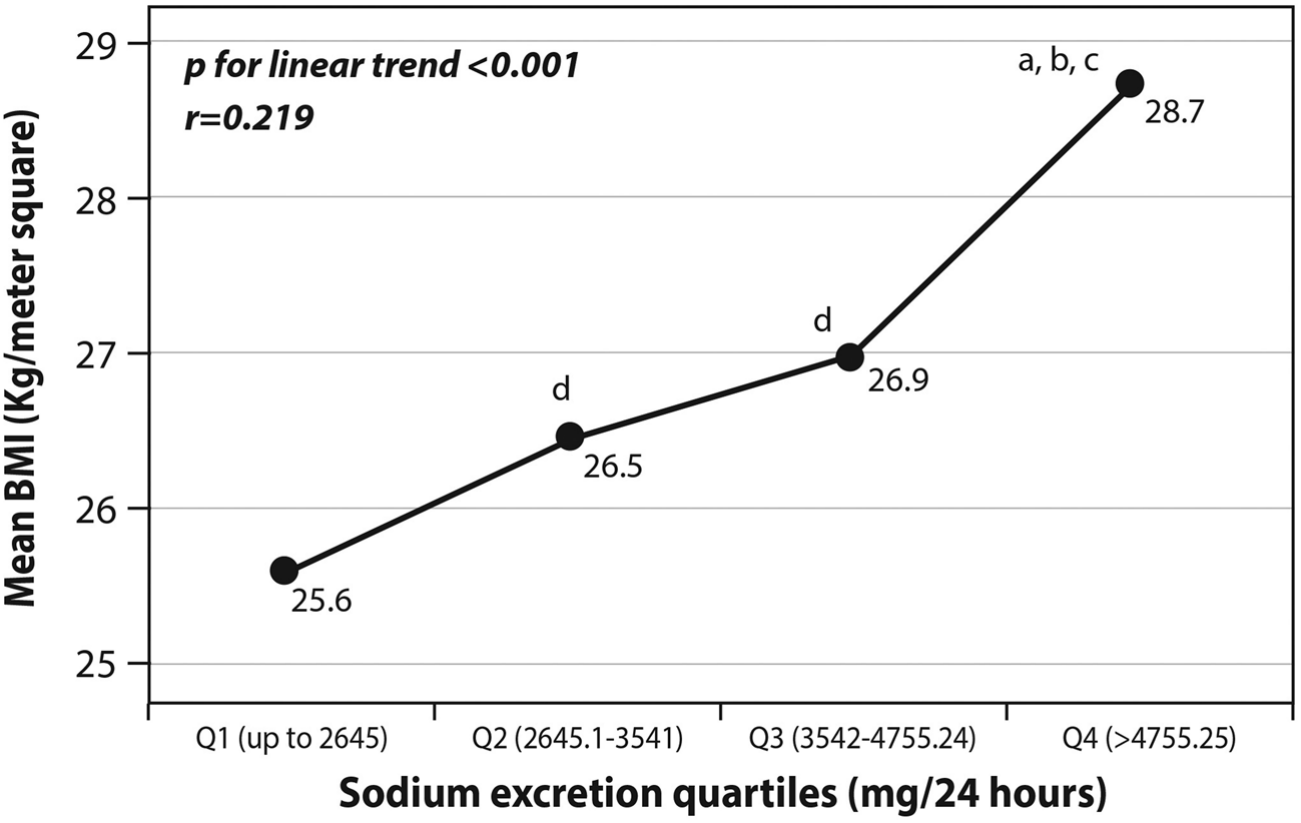
univariate associations between sodium excretion quartiles and mean BMI. Statistical differences between sodium excretion levels (analyzed using One-Way Analysis of variance [ANOVA]) are represented by the following letters: a = significantly different than Q1 (p < 0.05); b = significantly different than Q2 (p < 0.05); c = significantly different than Q3 (p < 0.05); d = significantly different than Q4 (p < 0.05).

**Figure 5:**
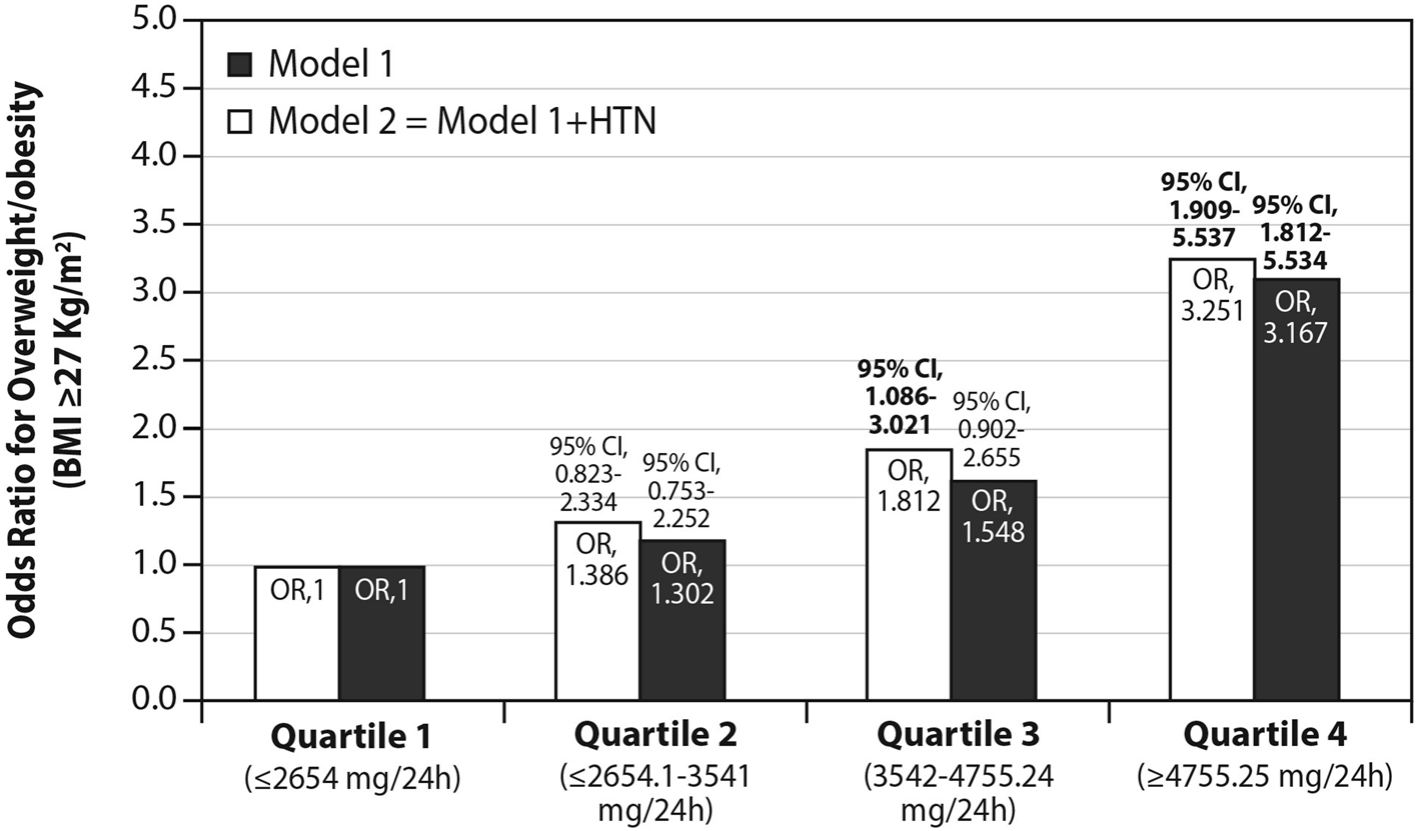
The independent association between sodium excretion and odds for overweight/obesity. Results from a multivariate logistic regression: Model 1 (solid bars)—represents adjustment to other explanatory variables. Model 2 (open bars) represents model 1 + adjustment to measured hypertension (≥ 140 and/or 90 mm/Hg). As compared to the lowest level (Q1), the highest level of sodium excretion (represented as Q4) was significantly and positively associated with higher odds for overweight/ obesity in both models. Adjustment for: the presence of diabetes (0 = no; 1 = yes), coronary heart disease (0 = no; 1 = yes), age (up to 43.9 = 0; 44–65 = 1), , ideal physical activity (As recommended by the American College of Sports Medicine [ACSM]-150 weekly minutes or more of moderate intensity or 75 weekly minutes of intense physical activity; 0 = no; 1 = yes) and sodium excretion quartiles (first quartile = reference]) and sex (0 = female; 1 = male) + (in model 2 only) measured hypertension (≥ 140 and/or 90 mm/Hg).

Sodium excretion was related to BMI (Table 2; Fig. 4), such that it increased according to BMI categories from the lowest mean levels in those with normal BMI to the highest excretion in obese subjects (Table 2; P for linear trend < 0.01). Likewise, with rising quartiles of sodium excretion, mean BMI also increased (p < 0.001 for trend; Fig. 4). Also, the OR for the presence of increased weight or obesity (BMI ≥ 27 kg/m^2^) was > 3 folds higher in interviewees whose sodium consumption was at the upper quartile, after adjustment for confounders (hypertension, age, sex, diabetes, CHD, physical activity; Fig. 5).

#### Sodium excretion according to the presence of overweight/obesity and hypertension

**Figure 6:**
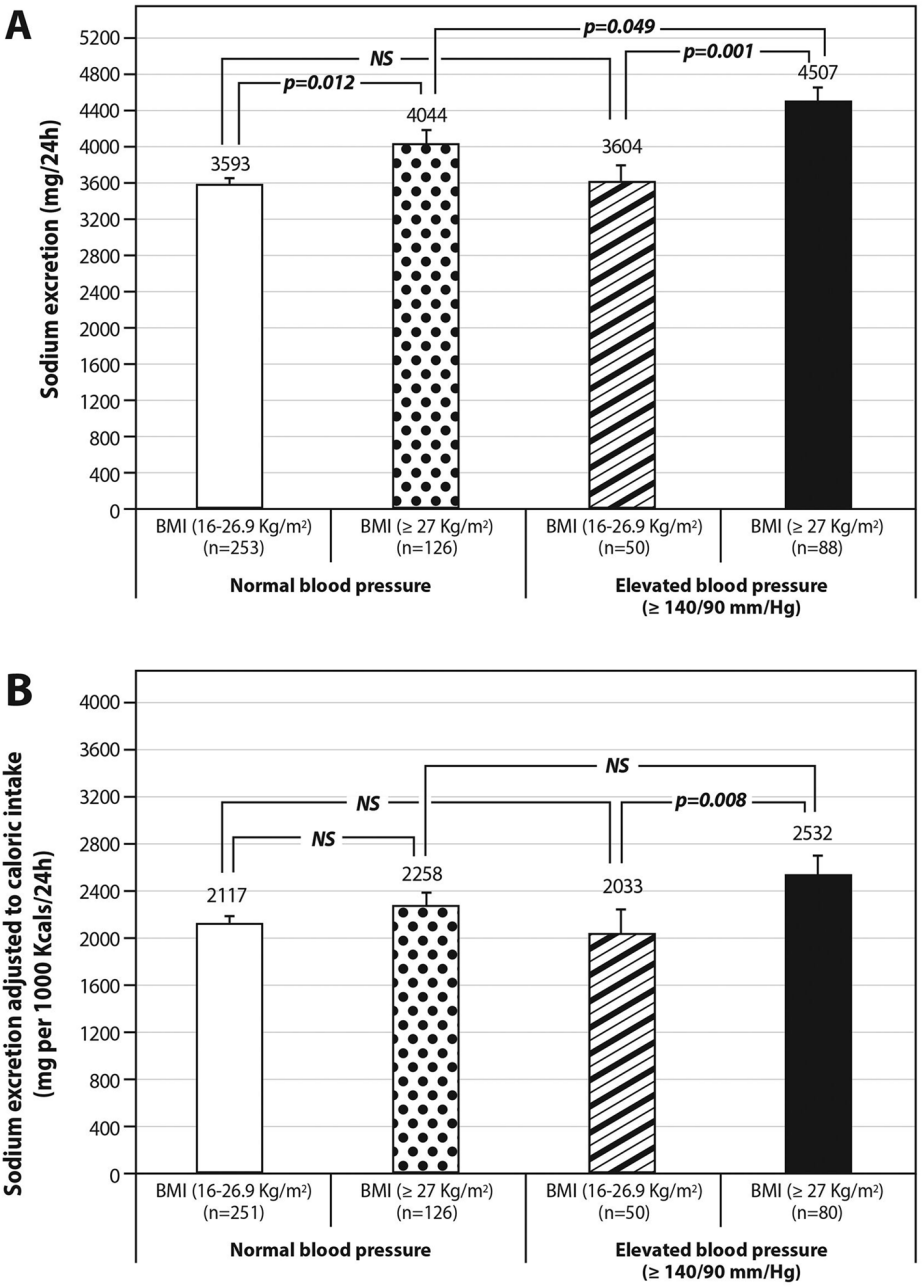
(**A**,**B**) Sodium excretion stratified by measured hypertension and overweight/ obesity status. 24-h sodium excretion (**A**) and 24-h sodium excretion density (excretion per 1000 Kcals) (**B**) among 4 combined phenotypes of hypertension and BMI: (1) normotensive + non-obese; (2) normotensive + overweight/obese; (3) hypertensive + non-obese; (4) hypertensive + overweight/obese. The different excretion levels of sodium were compared across each two pairs of phenotypes using independent t-test. Error bars represented the standard errors.

Sodium excretion was the highest in overweight/obese hypertensive (blood pressure ≥ 140/90 mmHg) interviewees (4507 ± 1656 mg) [p = 0.001]; compared to non-obese hypertensive (3604 ± 1306 mg) or normotensive interviewees (3593 ± 1604 mg); [p < 0.0001] and compared to overweight/obese normotensive interviewees (4044 ± 1725 mg; p = 0.049) (Fig. 6A). Likewise, the highest sodium density was found in hypertensive subjects who were also overweight or obese, but the difference was statistically significant only when compared to non-obese hypertensive subjects (Fig. 6B). Similar results are seen if the interviewees are categorized into non-obese vs. obese (less than or ≥ 30 kg/m^2^) (Figure S4).

## Discussion

The first key finding in this study is that in the current nutritional environment, sodium excretion, and by inference, salt intake, is excessive and clearly linked to overall caloric intake. Bread, vegetables, including commercially prepared/cooked vegetables, cheese and poultry were the main sources of sodium, but they collectively accounted for less than half of the dietary salt, thus indicating that dietary sodium is derived from diverse sources.

The link between overall caloric intake and sodium excretion was independent of potential confounders, and, notably, independent of BMI, an important factor in sodium excretion here and in previous reports^19^. Increasing caloric intake with higher sodium excretion was found in both normotensive and hypertensive interviewees. The large sex-related excess in salt excretion in men relative to women was annulled after adjustment for caloric intake. Finally, the association of higher urinary sodium excretion with hypertension was markedly weakened (but remained robust) once adjusted for caloric intake.

The link between caloric and sodium intake might be intuitive but is not trivial and requires targeted research. Obviously, high salt levels are present in many foods, including those with high caloric value. Additionally, both salted food or increased caloric intake may elicit mutual augmentation of consumption through the induction of general appetite and/or salt preference but the evidence for such interactions is inconsistent^20,21^. Other mechanisms may play a role, such as the co-presence in high calorie/high salt products of artificial sweeteners or yet other unsuspected "innocent bystanders", which may promote caloric intake^22^ or the induction of leptin resistance by high salt intake^23^. The persistence of high salt consumption in Western countries such as seen here, suggests that traditional professional advice to add less salt or refrain from salty foods^24^ is unlikely to have sufficient impact: the multitude of salt sources in food (Figure S2) comprises a serious limitation. Because salt and calories are clearly linked, caloric reduction should be tested as a potential additional tool.

The second key finding in this report is that when the interviewees were categorized according to the presence of both hypertension and overweight/obesity, as complex phenotypes, the highest consumption of salt was seen in overweight/obese hypertensive subjects. While our data are entirely concordant with cumulative data suggesting both obesity and hypertension are linked to high salt intake^25–29^, we emphasize that the combined obese-hypertensive phenotype stands out, in terms of excess salt consumption, even after adjustment for caloric intake.

**Figure 7:**
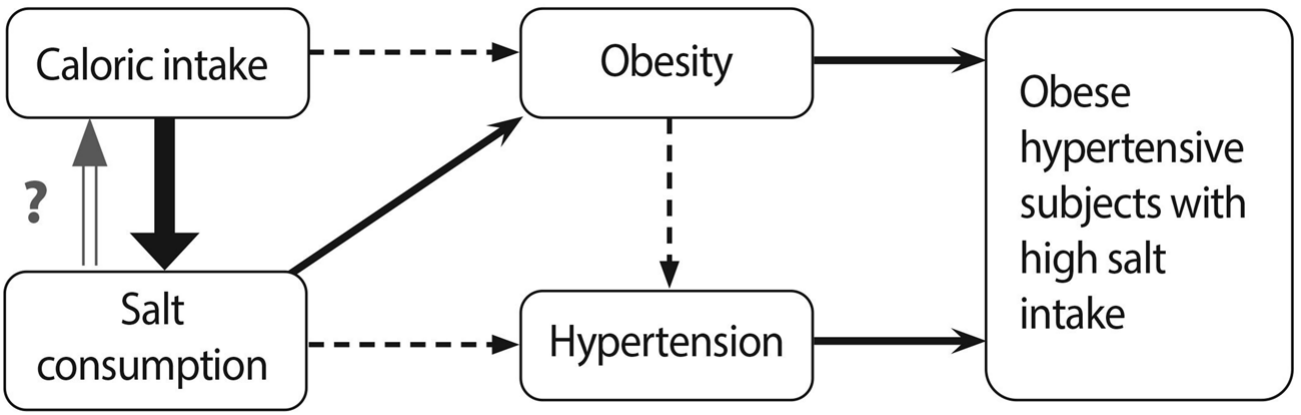
Proposed model of the relationship between caloric intake, salt consumption, hypertension and obesity based on the present survey and existing concepts. Key findings from this study are marked by a bold arrow and confirmatory findings by regular arrows. These are integrated into established connections (dashed arrows). Overall caloric intake promotes not only obesity, but also salt intake which is independently linked to hypertension and obesity. Obese hypertensive subjects consume, on the average, more salt than other phenotypes, when the presence/absence of obesity and hypertension are considered.

The sodium excretion data vis-a-vis sodium intake, caloric intake, hypertension and obesity can be therefore summarized as follows: (1) sodium intake is linked to caloric intake; (2) excessive sodium intake is quantitatively but separately related to blood pressure and overweight/obesity, independent of caloric intake; (3) high salt intake is observed particularly in overweight and obese subjects with hypertension. This group may then comprise the target population for increased efforts to reduce salt consumption. Since sodium excretion and caloric intake are linked, the possibility that lowering overall caloric intake may help reduce sodium intake merits consideration. Figure 7 summarizes this concept.

The strengths of the study include a large sample of Israeli adults, distributed country-wide with urine samples collected on weekdays and weekends with representation of all seasons; detailed dietary intake data with subsequent enablement of calibration of dietary intake tools with the "gold standard" measurement of sodium intake, based on 24hNa. This survey is unique in that it excluded subjects using drugs that potentially affect sodium excretion. Although the chronic use of such drugs likely achieves a stable and new balance between intake and excretion, incomplete compliance, recent onset of the use of these drugs and the diseases for which these drugs were prescribed and a secondary drug-induced rise in sodium intake to compensate for exaggerated sodium loss could introduce unwanted bias.

Limitations: The data apply only to the 20–65 years age group, with some under-representation of the 20–34 age group, and over-representation of the 55 + age group. There is an unplanned under-representation of Arabs. Finally, assurance of completeness of collection is impossible to attain.

### Conclusions

In the current nutritional environment overall caloric intake per se may be a driving force in salt consumption, but reverse causation cannot be excluded. The highest sodium excretion is seen among overweight/obese hypertensive interviewees. We suggest that (a) hypertension in obese subjects' merits particular targeting with respect to salt consumption; (b) overall caloric restriction be tested as a potential ancillary tool in the pursuit of reducing salt intake.

## Supplementary Information

Supplementary Information.

## Data Availability

The datasets used and/or analyzed during the current study are available from the corresponding author on reasonable request.

## References

[1] Yang Q, Liu T, Kuklina EV, Flanders WD, Hong Y, Gillespie C (2011). Sodium and potassium intake and mortality among US adults: Prospective data from the third national health and nutrition examination survey. Arch. Intern. Med..

[2] Mozaffarian, D., Fahimi, S., Singh, G. M., Micha, R., Khatibzadeh, S., Engell, R. E. *et al*. Global sodium consumption and death from cardiovascular causes. 10.1056/NEJMoa1304127 (2014). 10.1056/NEJMoa1304127 25119608

[3] Appel LJ (2003). Effects of comprehensive lifestyle modification on blood pressure control: main results. JAMA.

[4] Tanaka H, Tanaka Y, Hayashi M, Ueda Y, Date C, Baba T (1982). Secular trends in mortality for cerebrovascular diseases in Japan, 1960 to 1979. Stroke.

[5] Cao J, Eshak ES, Liu K, Gero K, Liu Z, Yu C (2019). Age-period-cohort analysis of stroke mortality attributable to high sodium intake in China and Japan. Stroke.

[6] Whelton PK, Appel LJ, Sacco RL, Anderson CAM, Antman EM, Campbell N (2012). Sodium, blood pressure, and cardiovascular disease. Circulation.

[7] World Health Organization. Guideline: sodium intake for adults and children [Internet] Geneva: World Health organization [Internet]. 2012. Available from: https://www.who.int/nutrition/publications/guidelines/sodium_intake/en/. 23658998

[8] McLean RM (2014). Measuring population sodium intake: A review of methods. Nutrients.

[9] Jürgens G (2018). Sodium excretion in population subgroups. JAMA.

[10] Chen, S. L., Dahl, C., Meyer, H. E. & Madar, A. A. Estimation of salt intake assessed by 24-hour urinary sodium excretion among Somali adults in Oslo, Norway. Nutrients [Internet]. 2018 Jul 13 [cited 2019 Jul 1];10(7). Available from: https://www.ncbi.nlm.nih.gov/pmc/articles/PMC6073275/. 10.3390/nu10070900 PMC6073275 30011847

[11] Land M-A, Neal BC, Johnson C, Nowson CA, Margerison C, Petersen KS (2018). Salt consumption by Australian adults: A systematic review and meta-analysis. Med. J. Aust..

[12] Cogswell ME, Loria CM, Terry AL, Zhao L, Wang C-Y, Chen T-C (2018). Estimated 24-hour urinary sodium and potassium excretion in US adults. JAMA.

[13] Rosenberg E, Lev B, Bin-Nun G, McKee M, Rosen L (2008). Healthy Israel 2020: A visionary national health targeting initiative. Public Health.

[14] Webster J, Dunford E, Hawkes C, Neal B (2011). Salt reduction initiatives around the world. J. Hypertens..

[15] insurance in the Health Maintenance Organizations in Israel-report by the social security organization 2016 [Internet]. Available from: https://www.btl.gov.il/Publications/survey/Pages/seker_289.aspx.

[16] von Elm E, Altman DG, Egger M, Pocock SJ, Gøtzsche PC, Vandenbroucke JP (2007). The strengthening the reporting of observational studies in epidemiology (STROBE) statement: Guidelines for reporting observational studies. Ann. Intern. Med..

[17] Dunsky A, Zach S, Zeev A, Goldbourt U, Shimony T, Goldsmith R (2012). Prediction of standing height among Israeli older adults: Results from a national survey. Ann. Hum. Biol..

[18] Tzameret Dietary Analysis software [Internet]. 2016. Available from: https://www.health.gov.il/Subjects/FoodAndNutrition/Nutrition/professionals/Pages/Tzameret.aspx.

[19] Moosavian SP, Haghighatdoost F, Surkan PJ, Azadbakht L (2017). Salt and obesity: A systematic review and meta-analysis of observational studies. Int. J. Food Sci. Nutr..

[20] Bolhuis DP, Lakemond CMM, de Wijk RA, Luning PA, de Graaf C (2012). Effect of salt intensity in soup on ad libitum intake and on subsequent food choice. Appetite.

[21] Zhang, Y., Li, F., Liu, F.-Q., Chu, C., Wang, Y., Wang, D. *et al*. Elevation of fasting ghrelin in healthy human subjects consuming a high-salt diet: A novel mechanism of obesity? Nutrients [Internet]. 2016 May 26 [cited 2019 Sep 28];8(6). Available from: https://www.ncbi.nlm.nih.gov/pmc/articles/PMC4924164/. 10.3390/nu8060323 PMC4924164 27240398

[22] Hill SE, Prokosch ML, Morin A, Rodeheffer CD (2014). The effect of non-caloric sweeteners on cognition, choice, and post-consumption satisfaction. Appetite.

[23] Lanaspa MA, Kuwabara M, Andres-Hernando A, Li N, Cicerchi C, Jensen T (2018). High salt intake causes leptin resistance and obesity in mice by stimulating endogenous fructose production and metabolism. Proc. Natl. Acad. Sci. USA.

[24] Salt reduction [Internet]. WHO; [cited 2020 Oct 10]. Available from: https://www.who.int/news-room/fact-sheets/detail/salt-reduction.

[25] Jackson SL, Cogswell ME, Zhao L, Terry AL, Wang C-Y, Wright J (2018). Association between urinary sodium and potassium excretion and blood pressure among adults in the United States: National Health and Nutrition Examination Survey, 2014. Circulation.

[26] Yoon YS, Oh SW (2013). Sodium density and obesity; The Korea National Health and Nutrition Examination Survey 2007–2010. Eur. J. Clin. Nutr..

[27] Hoffmann IS, Cubeddu LX (2009). Salt and the metabolic syndrome. Nutr. Metab. Cardiovasc. Dis..

[28] Ellison RC, Sosenko JM, Harper GP, Gibbons L, Pratter FE, Miettinen OS (1980). Obesity, sodium intake, and blood pressure in adolescents. Hypertens. Dallas Tex. 1979.

[29] Ma Y, He FJ, MacGregor GA (2015). High salt intake. Hypertension.

