# Supplementary material for: The role of caloric intake in the association of high salt intake with high blood pressure
Source: Sci Rep. 2021 Aug 4;11:15803. doi: 10.1038/s41598-021-95216-y (PMC8339119; doi:10.1038/s41598-021-95216-y)
Supplement: Supplementary file 1 — Supplementary Information. [file 41598_2021_95216_MOESM1_ESM.docx]

**DATA SUPPLEMENT**

**The role of caloric intake in the association of high salt intake with high blood pressure**

**Naftali Stern****^1,2^, M.D., Assaf Buch^1,2^, Ph.D., Rebecca Goldsmith^3^, MPH., Lesley Nitsan^3^, MAN., Miri Margaliot^1^, M.H.A., Ronit Endevelt^3,5^, Ph.D., Yonit Marcus^1,2^,** **MD., Ph.D., Gabi Shefer^1^, Ph.D., and Itamar Grotto^4,6^,** **MD., Ph.D.**

1. The Sagol Center for Epigenetics of Aging and Metabolism, Institute of Endocrinology, Metabolism and Hypertension, Tel Aviv-Sourasky Medical Center; Sackler Faculty of Medicine, Tel Aviv University, Israel
2. The Sackler Faculty of Medicine, Tel-Aviv University, Tel-Aviv, Israel
3. Nutrition Department, Ministry of Health, Jerusalem, Israel
4. Public Health Services, Ministry of Health, Jerusalem, Israel
5. School of Public Health, University of Haifa, Haifa, Israel
6. Faculty of Health Sciences, Ben-Gurion University of the Negev, Be'er Sheva, Israel

ORCiD number: https://orcid.org/0000-0002-5123-3176 (N Stern)

**Short title:** Salt consumption is linked to caloric intake

**Address for correspondence and reprints:**

Naftali Stern, MD

The Sagol Center for Epigenetics of Metabolism and Aging

Institute of Endocrinology, Metabolism and Hypertension

Tel Aviv Sourasky Medical Center

Sackler Faculty of Medicine

Tel Aviv University, Israel

Tel: 972-3-6973732 ; Fax: 972-3-6973035

e-mail: naftalis@tlvmc.gov.il

Table of Contents

[Supplementary Method -1: Urine collection protocol 3](#_Toc52455428)

[Supplementary Method -2: Survey questionnaire 4](#_Toc52455429)

[Supplementary Method -3: Food Frequency Questionnaire (FFQ) 14](#_Toc52455431)

[Supplementary Method -4: Urine handling 17](#_Toc52455432)

[Supplementary Method -5: Data completion 17](#_Toc52455433)

[Supplemental Online Figures 18](#_Toc52455434)

# Supplementary Method -1: Urine collection protocol

Two-liter plastic bottles for urine storage, urine cups, insulated opaque carry bag, disposable gloves) were supplied. Urine collection was to commence after the first morning urination, and continue for a full 24 hours, with all urine to be collected, and stored in plastic bottles supplied for that purpose.

# Supplementary Method -2: Survey questionnaire

***Questionnaire*** ***no. ______ Interviewee No. ______ Interview Date: ___________ Start time________***

**Ministry of Health**

**Public Health Services Nutrition Department**

**Tel Aviv Sourasky Medical Center Institute for Endocrinology, Metabolism and hypertension**

**General Questionnaire, Sodium urine survey and FFQ**

**SALT INTAKE HABITS**

**Interviewer, read: The following questions relate to your eating habits, with the emphasis on salt intake.**

***(Interviewer: Do not read the answers “don’t know’ ‘refuse”, but mark them as needed).***

**3.1 Do you add salt to food at the table?**

1. Yes, always

2. Yes, sometimes

3. Usually not

4. Never

**3.2 For the food you eat at home, is salt added during cooking?**

1. Never

2. Rarely

3. Yes, sometimes

4. Often

5. Always

*8. Don’t know 9. Refuse*

**3.3 In your opinion, how much salt do you usually use?**

1. A large amount

2. Quite a lot

3. The desired amount

4. A little

5. Very little

*8. Don’t know 9. Refuse*

**3.4 In your opinion, can a high salt diet cause serious medical problems?**

1. Yes go to Q.3.5

2. No go to Q 3.6

*8. Don’t know* go to Q 3.6 *9. Refuse* go to Q 3.6

**3.5 What problem?** *More than one answer can be marked*

1. Hypertension

2. Osteoporosis

3. Stomach Cancer

4. Kidney Stones

5. None of the above

6. Each of the above

*8. Don’t know 9. Refuse*

**3.6 How important is it to you to reduce the amount of salt/sodium in your diet?**

1. Not important at all

2. Important to a certain extent

3. Very important

*8. Don’t know 9. Refuse*

**3.7 Do you take any action on a routine basis to limit your salt/sodium intake?**

1. Yes go to Q3.8

2. No go to q 3.9

*8. Don’t know* go to Q 3.9 *9. Refuse* go to Q 3.9

**3.8 What action do you take?** *More than one answer can be marked*

**1.** Reduce/abstain from eating processed foods

2. Check the sodium/salt content on the label of packaged food

3. Don’t add salt to food at the table

4. Buy foods that are salt free or salt-reduced

5. Buy low sodium foods

6. Don’t add salt during cooking

7. Use other spices instead of salt during cooking

8. Avoid eating out

9. Other, specify: _____________________________________________

**3.9 Which salt do you usually add at the table?** *More than one answer can be marked*

1. Don’t add at all

2. “Regular” salt, including seasoned (not sea salt, not iodized salt)

3. Sea salt

4. Iodized salt

5. Salt substitute (potassium- based) name: _____________

*8. Don’t know 9. Refuse*

**3.10 Which salt do you usually add in cooking?** *More than one answer can be marked*

1. Don’t add at all

2. “Regular” salt, including seasoned (not sea salt, not iodized salt)

3. Sea salt

4. Iodized salt

5. Salt substitute (potassium- based) name: _____________

*8. Don’t know 9. Refuse*

**3.11 Do you use the following spices/herbs or other spices during cooking and /or add at the table?**

| **Citric acid** | **Chicken seasoning** | **Pizza seasoning** | **Fish seasoning** | **Steak seasoning** |
| --- | --- | --- | --- | --- |
| 1. Yes  2. No | 1. Yes  2. No | 1. Yes  2. No | 1. Yes  2. No | 1. Yes  2. No |

**3.12 Do you use soup powders or sauces while cooking?**

1. Yes, often

2. Sometimes

3. Rarely *go to Q3.14*

4. Not at all *go to Q3.14*

**3.13 Which powders/sauces do you usually use?**

**1._________________________________________________**

**2. __________________________________________________**

**3. ___________________________________________________**

**3.14 Do you keep kosher? (Jews only)**

1. Yes, always

2. Yes, only at home

3. Other, specify: __________________________________________

4. No

**3.15 Is the beef you buy always kosher?**

1. Yes, always

2. Sometimes

3. Usually not

4. Never

*8. Don’t know 9. Refuse*

**3.16 Is the chicken/turkey you buy always kosher?**

1. Yes, always

2. Sometimes

3. Usually not

4. Never

*8. Don’t know 9. Refuse*

**4. QUESTIONS ON HEALTH STATUS, LIFESTYLE, AND HEALTH HABITS**

**4.1. Has a doctor ever diagnosed you with?**

|  | **Disease** | **Yes, on medication** | **Yes, not on medication** | **No** | **Don’t know** | **Refuse** |
| --- | --- | --- | --- | --- | --- | --- |
| 4.1.1 | Hypercholesterolemia | 1 | 2 | 3 | 4 | 5 |
| 4.1.2 | Hypertriglyceridemia | 1 | 2 | 3 | 4 | 5 |
| 4.1.3 | Diabetes | 1 | 2 | 3 | 4 | 5 |
| 4.1.4 | Heart Disease | 1 | 2 | 3 | 4 | 5 |
| 4.1.5 | Stroke | 1 | 2 | 3 | 4 | 5 |
| 4.1.6 | Fatty Liver | 1 | 2 | 3 | 4 | 5 |
| 4.1.7 | Hypertension | 1 | 2 | 3 | 4 | 5 |

| **4.2 When you read the information on the nutrition label, do you check?** |  |
| --- | --- |
| 4.2.1 Nutrition claims, such as: low fat, lite, low sodium | 1. Always  2. Often  3. Rarely  4.Never |
| 4.2.2 The amount of sodium/100 gram food | 1. Always  2. Often  3. Rarely  4.Never |

**4.3 Do you now smoke, including a hookah?**

1. Yes *go to question 4.4*

2. Not current, I used to smoke in the past *go to question 4.6*

3. No, I have never smoked *go to question 4.6*

**4.4 What do you currently smoke / smoked in the past?** *Mark all possible answers*

1. Cigarettes

2. Cigars *go to question 4.6*

*3.* A pipe *go to question 4.6*

4. A hookah *go to question 4.6*

5. Other, specify: _______________________________ *go to question 4.6*

**4.5 How many cigarettes a day/week do you smoke or did you smoke? ____ cigarettes per day /week**

**4.6. During a regular week, during leisure hours, do you regularly engage in physical activity? Such as walking, running, swimming, sport, ball games, lasting at least 20 consecutive minutes? ‘**

1. Yes

2. No ***go to question 4.8***

**4.7. During a regular week, how often do you engage in physical activity, including all types?**

1. 5 times a week or more

2. 4 times a week

3. 3 times a week

4. 1-2 times a week

5. Less than once a week

**4.8 During a regular week, do you regularly engage in strenuous physical activity for at least 10 consecutive minutes? Meaning activity which causes profuse sweating or increase in breathing or heart rate (for example: running, fast walking, aerobic activity, etc.)**

1. Yes

2. No ***go to question 4.11***

**4.9 During a regular week, how often do you engage in this strenuous physical activity?**

________ times 1. Daily 2. Weekly 222. Refuse 333. Don’t know

**4.10 During a regular week, how much time, in total, do you engage in this strenuous physical activity?**

________ minutes ________ hours 222. Refuse 333. Don’t know

**4.11 During a regular week, have you regularly engaged in moderate physical activity for at least 10 consecutive minutes? Meaning activity which causes mild sweating or a small increase in breathing or heart rate (for example: walking at a moderate pace, swimming at a slow pace, etc.)**

1. Yes

2. No ***go to question 4.14***

**4.12 During a regular week, how often do you engage in this moderate physical activity?**

________ times 1. Daily 2. Weekly 222. Refuse 333. Don’t know

**4.13 During a regular week, how much time, in total, do you engage in this moderate physical activity?**

________ minutes ________ hours 222. Refuse 333. Don’t know

**4.14 Was yesterday (the day of the urine collection) a regular day for you, from the point of view of physical activity?**

1. Yes, the same

2. No, I did less than usual

3. No, I did more than usual

*8. Don’t know 9. Refuse*

**5. DEMOGRAPHIC QUESTIONS**

**5.1. How old are you?** _______

**5.2 Where were you born?**

1. Israel 2. Other country, specify: _______________

**5.3 What is your family status?**

1. Single

2. Married or living with a partner

3. Divorced or separated

4. Widowed

5. Other, specify: ________________

**5.4. Are you?**

1. Jewish

2. Christian (not Arab)

3. Arab Moslem

4 Arab Christian

5. Druze

6. Other, specify: ________________________

**5.5. Do you define yourself as a:**

1. Secular

2. Traditional

3. Religious

4. Ultra orthodox

5. Other, specify: _______________

**5.6** How **many years in total have you studied in all schools, colleges, including elementary school and trade studies (not including incomplete years)? ________**

**5.7 What is the highest degree you have?**

1. Matriculation/high school certificate

2. Professional /trade certificate, without matriculation

3. Professional /trade certificate, with matriculation

4. First academic degree

5. Second academic degree

6. Third academic degree

7. Rabbinic degree or from a Yeshiva

8. Other certificate, specify: _____________

9. No certificate

**5.8. How many people live in the apartment (house) you live in?( not including a tenant, a soldier living away from home, student in dormitories or living away from home) _______**

**5.9. How many rooms are there in the apartment/house you live in (not including the kitchen, bathrooms and rooms used solely for business purposes, or for a tenant)? _____**

**5.10 What is your total net monthly income, from all sources, after deduction of income tax, pension funds, and other insurances?**

1. Up to 2000 NIS
2. 2001 – 3000 NIS
3. 3001 – 4000 NIS
4. 4001 – 5000 NIS
5. 5001 – 6000 NIS
6. 6001 – 7500 NIS
7. 7501 – 10,000 NIS
8. 10,001 – 14,000 NIS
9. Above 14,001 NIS
10. (Not relevant-for example: lives on a kibbutz, non-working student, collective settlement)

222. Refuses

**6. MEDICATIONS AND SUPPLEMENTS INTAKE**

**6.1 Do you take medications or nutrition supplements (including vitamins) on a regular basis?**

1. Yes

2. No ***go to the next section***

**6.2 Could you please bring all the medications and/ or nutrition supplements (including vitamins) you take on a regular basis, taken by mouth, by injection or as suppositories?**

1. Yes

2. No, I am not prepared to bring them ***Go to the next section “Measurements”***

***Interviewer: List each medication and supplement- make sure to write in English in clear capital letters. Copy the name from the packet. Write each one individually on a separate line and ask about the reason and dose.***

| **Drug/**  **supplement name** | **Manufacturer** | **Purpose for taking the medication/supplement** | **Dose ( no. of units each time)** | **No. of times** | **Time period** |
| --- | --- | --- | --- | --- | --- |
| 1. |  | 1. __________  2. Don’t know |  |  |  |
| 2. |  | 1. __________  2. Don’t know |  |  |  |
| 3. |  | 1. __________  2. Don’t know |  |  |  |
| 4. |  | 1. __________  2. Don’t know |  |  |  |
| 5. |  | 1. __________  2. Don’t know |  |  |  |
| 6. |  | 1. __________  2. Don’t know |  |  |  |

**7. MEASUREMENTS**

| **Height (cm)**  1. _____________ ***Measured with shoes*** 1. Yes 2. No  2. _______________  3. _______________________  4. Not measured/ unable to measure | **Weight (kg)**  1. __________ ***Weighted with shoes*** 1. Yes 2. No  2. _______________  3._______________________________  4. Not weighted/ unable to weigh |
| --- | --- |

**Blood pressure measurements**

| **1. Systolic blood pressure** | **2. Diastolic blood pressure** | **3. Pulse** |
| --- | --- | --- |
| 1.1________ | 2.1________ | 3.1________ |
| 1.2________ | 2.2________ | 3.2________ |
| 1.3________ | 2.3________ | 3.3________ |

**If not measured, what is the reason? ________________________________________________________________**

**Urine collection data:**

**Amount (ml)**

**Bottle 1 (2 liter bottle)___________________**

**Bottle 2 (2 liter bottle)___________________**

**Start time of urine collection: ____________________________**

**End time of urine collection: ____________________________**

**Time of SPOT sample: ____________________________**

**Were there problems?**

1. **Yes 2. No**

**Comments:_______________________________________________________________________________________________________________________________________________________________________________________________________________________________________________**

***Interviewer –after you are seated, and ready to begin, read*: “**Hello, my name is _______. Thank you for your willingness to answer some questions regarding your state of health and your eating habits. If, at any stage during the interview, any of the questions are not clear, please tell me. If you wear glasses for reading, please bring them.

We will begin the interview with some questions about what you ate and drank yesterday.

**1. 24-hour food recall**

| **What did you eat from 4 a.m. yesterday _____until 4 a.m.** **today____?** ***If necessary, read*** “Previous studies have shown that at 4:00 am. it is possible to distinguish between one day of 24 hours and the next.”  Specify everything you ate and drank, in the house and outside of the house, at mealtimes and in between, including sweets and snacks, coffee, tea soft drinks, alcoholic drinks etc.  ***Interviewer: Write each item During a separate row*** | |  | **What time did you begin to eat /drink the item?** |  |
| --- | --- | --- | --- | --- |
|  | **The quick list** | **√** |  | ***CARD 1*** |
| A |  |  |  | **Where did you eat?** |
| B |  |  |  | 1. At home (home cooked food) |
| C |  |  |  | 2. Home (ready made, /bought |
| D |  |  |  | food |
| E |  |  |  | 3. Dining room (sheltered |
| F |  |  |  | housing ) |
| G |  |  |  | 4. Restaurant |
| H |  |  |  | 5. Day Care Center for the Elderly |
| I |  |  |  | 6. At work (home-prepared food) |
| J |  |  |  | 7. At work( ready made/bought |
| K |  |  |  | Food) |
| L |  |  |  | 8. At work (cafeteria, dining room) |
| M |  |  |  | 9. Other, specify:____________ |
| N |  |  |  |  |
| O |  |  |  | ***CARD 1*** |
| P |  |  |  | **Which meal was it?** |
| Q |  |  |  | 1. Before breakfast |
| R |  |  |  | 2. Breakfast |
| S |  |  |  | 3. Mid-morning snack |
| T |  |  |  | 4. Lunch |
| U |  |  |  | 5. Mid-afternoon snack |
| V |  |  |  | 6. Dinner |
| W |  |  |  | 7. Late night snack |
| X |  |  |  | 8. Undefined meal/drink (snack) |
| Y |  |  |  | 9. Food/ consumed over several |
| Z |  |  |  | hours |
|  |  |  |  | 10. Other ,specify:_______________ |

***To the interviewer - read: “*** There are foods which people forget they ate or drank, or they ate them without being aware. Try to remember if you forgot to mention one of these foods: hot drinks, cold drinks (including water), alcoholic drinks, sweets, salty snacks, fruits, vegetables, bread”.***To the interviewer - read:*** “I would now like to ask you for additional details regarding the food and drinks you mentioned. I will ask where you ate and if this item was part of a meal. If you remember something else, tell me. When I ask you about the quantity you ate or drank, you can use the examples I suggest, the dishes in your house or the information on the wrapper/packet”.

**Food/Drink description.**

***To the interviewer – Transfer, from the Quick List the item letter and hour to columns 1 and 2, and item name to column 5. Mark √ on the Quick List in the column next to the item you’ve copied and go on to complete columns 3 and 4 using Card 1, and afterwards, complete columns 6 and 7 using the questions in the “Food and Food Quantities Guide”.***

| **Item**  **Letter** | **Hour** | **Where did you eat/drink this item?** | **Which meal was it?** | **Item name** | **Food/drink description** | **What quantity did you eat/drink?** |
| --- | --- | --- | --- | --- | --- | --- |
| **1** | **2** | **3** | **4** | **5** | **6** | **7** |
|  |  |  |  |  | 1 |  |
|  |  |  |  |  | 2 |  |
|  |  |  |  |  | 3 |  |
|  |  |  |  |  | 4 |  |
|  |  |  |  |  | 5 |  |
|  |  |  |  |  | 6 |  |
|  |  |  |  |  | 7 |  |
|  |  |  |  |  | 8 |  |
|  |  |  |  |  | 9 |  |
|  |  |  |  |  | 10 |  |
|  |  |  |  |  | 11 |  |
|  |  |  |  |  | 12 |  |
|  |  |  |  |  | 13 |  |
|  |  |  |  |  | 14 |  |
|  |  |  |  |  | 15 |  |
|  |  |  |  |  | 16 |  |
|  |  |  |  |  | 17 |  |
|  |  |  |  |  | 18 |  |
|  |  |  |  |  | 19 |  |
|  |  |  |  |  | 20 |  |
|  |  |  |  |  | 21 |  |
|  |  |  |  |  | 22 |  |
|  |  |  |  |  | 23 |  |
|  |  |  |  |  | 24 |  |
|  |  |  |  |  | 25 |  |
|  |  |  |  |  | 26 |  |
|  |  |  |  |  | 27 |  |
|  |  |  |  |  | 28 |  |
|  |  |  |  |  | 29 |  |
|  |  |  |  |  | 30 |  |
|  |  |  |  |  | 31 |  |
|  |  |  |  |  | 32 |  |
|  |  |  |  |  | 33 |  |
|  |  |  |  |  | 34 |  |
|  |  |  |  |  | 35 |  |
|  |  |  |  |  | 36 |  |
|  |  |  |  |  | 37 |  |
|  |  |  |  |  | 38 |  |
|  |  |  |  |  | 39 |  |
|  |  |  |  |  | 40 |  |
|  |  |  |  |  | 41 |  |
|  |  |  |  |  | 42 |  |
|  |  |  |  |  | 43 |  |
|  |  |  |  |  | 44 |  |
|  |  |  |  |  | 45 |  |

**1. Was the amount you ate yesterday similar to the amount you usually eat?**

1. Yes, the same ***go to next section (Section 2)***

2. No, yesterday I ate less than usual

3. No, yesterday I ate more than usual ***go to question 2***

**2. What is the main reason you ate a different amount yesterday to that you usually eat?**

1. Diet

2. Vacation, trip, travel

3. Lack of time

4. Religious holiday, Shabbat

5. Family celebration, social occasion

6. Stress, boredom

7. Illness, feeling unwell

8. A fast day

9. Other, specify: __________________

# Supplementary Method -3: Food Frequency Questionnaire (FFQ)

In order to assist the interviewee in identifying food types and quantities during the food recall, the interviewers used the “Food and Food Quantities Guide” ^28^, which was developed and produced in 2009 by the Israeli Ministry of Health. The Guide includes many photographs of Israeli foods as well as detailed questions (such as whether the food was purchased or prepared at home; the method of preparation: whether it was eaten with side dishes, the fat percentage of the food etc.), so as to characterize as accurately as possible the food/beverage consumed. During the interview, interviewers used cards, the “Food and Food Quantities Guide” ^28^ and measuring aids such as a measuring cup, a tablespoon and a teaspoon in order to facilitate quantification of amounts consumed.

**FFQ**

**Interviewer, read: I will now read you a list of foods and I will ask you, for each food that I mention, to tell me, according to the serve size, what is the daily, weekly or monthly amount that you ate in the past year.**

| **Serve size** | **Food** | **no** |
| --- | --- | --- |
| 1 cup | Milk 0-2%' including goats, sheep ( inc,. in coffee | 1 |
| 1 cup | Milk more than 2% (inc. in coffee) | 2 |
| 1 cup | Chocolate or other flavored milk | 3 |
| 1 spoon (1 container= 6 spoons | Cheese, white, or cream or cottage, up to 5% fat | 4 |
| 1 spoon (container= 6 spoons | Cheese, white, or cream or cottage, more than 5% fat | 5 |
| 1 container | Leben, yogurt 0-1.5% fat, without additions and/or sugar | 6 |
| 1 container | Leben, yogurt 3% fat or more, without additions and/or sugar, including sour cream | 7 |
| 1 container | Leben, yogurt, milk pudding 0-1.5% fat, with additions and/or sugar | 8 |
| 1 container | Leben, yogurt, milk pudding 3% fat or more, with additions and/or sugar | 9 |
|  |  | 10 |
| 1 slice, one triangle | Cheese, yellow or processed, 9% fat or less | 11 |
| 1 slice, one triangle | Cheese, yellow or processed, all types, more than 9% fat | 12 |
| 1 slice or 10 cubes | Cheese, salty, all types, 5% fat or less | 13 |
| 1 slice or 10 cubes | Cheese, salty, all types, more than 5% fat | 14 |
| 1 spoon | Labane, including Jamid, Kishk' or Labane balls | 15 |
| Cup or container | Soy drink or soy dessert/pudding | 16 |
| 1 egg | Egg-based recipes (fried, scrambled,omelet, shakshuka) | 17 |
| 1 egg | Egg, soft or hard boiled (yolk/white=1/2 egg) | 81 |
| Medium serve | Chicken or turkey schnitzel. Home-made or home style, bought | 19 |
| One serve | Shnitzels, nuggets, processed purchased | 20 |
| 1/4 chicken or a serve | Chicken or turkey (not schnitzel) | 21 |
| Medium serve | Beef, veal' including steak or goulash | 22 |
| One serve | Shawarma, shishlik in pita or lafa | 23 |
| 2 medium | Patties, from chicken, turkey, beef, includiong hamburger or kebab | 24 |
| 1 medium serve | Mixed dishes with beef, chicken or turkey, such as Kubbeh, stuffed vegetables, cholent, maklouba, maftoul, mansaf with beef, machmar. musachan | 25 |
| 1 medium serve | Internal organs of chicken or beef: liver, intestines, giblets, heart, tongue | 26 |
| 1 slice | Pastrama or smoked turkey breast | 27 |
| 1 unit or 2 slices | Sausage (salami) or sausages, all types | 28 |
| 1 medium serve | Fish, baked, fried, or cooked in sauce including patties | 29 |
| 1 heaped spoon | Tuna, canned including salad | 30 |
| 1 slice | Fish, cured, smoked, pickled, or canned, not including tuna | 31 |
| 1 unit | Meat substitutes- schnitzel,hamburger, sausages, soy kebab, soy patty, or other soy and tofu products | 32 |
| 1 unit | Vegetable schnitzel or patty | 33 |
| 1 medium serve | Mixed dishes without meat, including cholent, stuffed vegetables | 34 |
| 1 serve | Falafel in Pita | 35 |
| 1 slice or 1/2 roll/pita | Roll or pita or white bread or challah, including home baked | 36 |
| 1 slice or 1/2 roll/pita | Roll or pita or bread from whole wheat including rye and corn | 37 |
| 1 slice or 1/2 roll | Lite bread, lite challah or light rolls | 38 |
| **1 medium triangle** | Pizza | 39 |
| 1 slice, or medium unit | Pie or quiche with vegetables or cheese, crepes | 40 |
| 1 bureka/pastille/ jachnun or 1/2 melawach | Burekas, Melawach, Jachnun, pastilles | 41 |
| 1 cup | Cooked grains, not whole grain, such as pasta, noodles | 42 |
| 1 cup | Cooked grains, whole grain, such as pasta, noodles | 43 |
| 1 cup or medium serve | Cooked wheat, bulgur, couscous, quinoa, buckwheat, barley, corn | 44 |
| 1 cup | Rice, cooked white with or without sauce | 45 |
| 1 cup | Rice, cooked whole grain with or without sauce | 46 |
| 1 cup or medium serve | Potato, cooked or baked, including puree or potato salad | 47 |
| 1 medium serve | Potato, chips (not crisps) as a patty, latke | 48 |
| 1 bowl | Breakfast cereal, cooked , such as oatmeal, semolina | 49 |
| 1 medium bowl, I snack | Ready to eat breakfast cereal, such as cornflakes, granola, granola bar | 50 |
| 1/2 cup or portion | Cooked dried beans including soup, mixed cooked dishes (beans, fava beans) not including soy | 51 |
| 1 cup or medium serve | Fresh vegetable salad | 52 |
| 1 tomato, or 13 cherry | Tomatoes, or cherry tomatoes, cooked or fresh (if not in salad) | 54 |
| 1 tablespoon | Tomato sauce or ketchup | 55 |
| 1 cucumber | Cucumber, fresh or in salad (**if not mentioned)** | 56 |
| 1 pepper | Pepper, fresh or in salad (all colors) (**if not mentioned**) | 57 |
| 1 medium serve | Zucchini, eggplant cooked or baked or roasted | 58 |
| 1 medium serve | Broccoli, cauliflower, Brussel sprouts or mixed vegetables, cooked | 59 |
| 1 medium serve | Cabbage' white or red, including in fresh salad | 60 |
| 1 cup of lettuce | Fresh lettuce or other green leafy vegetables, including in salad | 61 |
| 1 carrot/1 cup juice | Carrot, fresh of cooked, including carrot juice | 62 |
| 1 cup | Peas, green or yellow beans, okra (including frozen, canned) | 63 |
| 1 medium serve | Sweet potato, pumpkin, butternut pumpkin | 64 |
| 1 cup or medium serve | Other vegetables including kohlrabi, mushrooms, and cooked leafy vegetables | 65 |
| 1 heaped tablespoon | Avocado (including avocado salad) | 66 |
| One serve | Prepared soup, such as Mana hama | 67a |
| One bowl | Soup with chicken or turkey or beef (if not mentioned)   1. **All year 2. Only in season** | 67 |
| One bowl | Vegetable soup (if not mentioned)  **All year 2. Only in season** | 68 |
| One bowl | Legume soup, or with barley or other (if not mentioned)  **1.All year 2. Only in season** | 69 |
| 1 heaped tablespoon | Hummus/Tehina salad (one serve of hummus has 8 tablespoons) | 70 |
| 1 heaped tablespoon | Cooked vegetable salads such as Turkish, Matboucha, eggplant- with or without mayonnaise | 71 |
| 1 unit | Pickled vegetables' **not including olives** | 72 |
| 10 olives | Olives | 73 |
| 1 medium | Apple/pear, fresh or baked or cooked (not including dried) | 74 |
| 1 medium or 1/2 grapefruit | **In season,** orange, grapefruit, mandarin or other citrus fruit | 75 |
| 1 medium | Banana | 76 |
| 1 medium | **In season**, peach, nectarine,plum, apricot ( not dried) | 77 |
| 12 pieces or handful | Grapes or sultanas/currants | 78 |
| 1 slice | **In season:** melon | 79 |
| 1 large slice / 2 small | **In season:** watermelon | 80 |
| 1/2 cup or one medium unit | Other fruit or fruit salad or cooked fruit | 81 |
| 1 cup | Fruit juice or frozen from citrus fruit or other | 82 |
| 4 pieces | Dried fruit (apricot, fig, prune, date, apple) | 83 |
| Small packet or 30 g or handful | Salty snacks such as: Bissli, potato crisps, pretzels, popcorn, soup nuts, croutons | 84 |
| 1 unit | Salty crackers such as lachmit, patit, rice cakes | 85 |
| 1 unit/ 2 scoops | Icecream- all milk-based types  **1.All year 2. Only in season** | 86 |
| 2 scoops | Icecream- non-dairy, including sherbet/sorbet  **1.All year 2. Only in season** | 87 |
| 1 ice lolly/popsicle, 2 scoops | Popsicle or sherbet type ice lolly or sorbet | 89 |
| 1/2 large snack bar or 1 mini or 1 row or 1 tablespoon | Milk or dark chocolate' or chocolate bar, all types ( including chocolate covered, wafer type) or chocolate spread | 90 |
| I unit | Cookies, plain type including all non-coated | 91 |
| I unit | Wafers and filled or coated cookies | 92 |
| I unit | Yeast cake or baked goods, such as rugelach, doughnuts | 93 |
| 1 slice | Plain cake, chocolate cake, Marble cake, fruit cake | 94 |
| 1 slice | Cheese cake or cream cake | 95 |
| I unit | Kanafe, kedayef' or baklawa or Halva | 96 |
| 1 teaspoon | Honey, jam, dulce la leche, chocolate spread, syrup | 97 |
| 25 g/handful | Salted Nuts, almonds, pistachios, salted peanuts | 98 |
| 25 g/handful | **Unsalted** Nuts, almonds, pistachios, salted peanuts | 98a |
| 1/2 cup | Sunflower/watermelon/pumpkin seeds | 99 |
| 1 teaspoon or 1 piece | Sugar, including in tea, coffee or lollies of all types | 100 |
| 1 cup | Carbonated beverage, not calorie reduced, including non-alcoholic beer | 101 |
| 1 cup | Carbonated/ non-carbonated, low calorie beverage | 102 |
| 1 cup | Fruit drink or fruit nectar' reduced energy ' or flavored water | 103 |
| 1 mug | Coffee, all types, including decaffeinated | 104 |
| 1 mug | Tea, all types | 105 |
| 1 can/ 1.5 cups | Beer all types, not including non-alcoholic | 106 |
| 1 wine glass | Wine | 107 |
| 1 shot glass | Alcoholic spirits | 108 |
| 1 cup | Tap water, including filtered water | 109 |
| 1 cup | Mineral water | 110 |
| 1 teaspoon | Butter, butter spread, ghee | 111 |
| 1 teaspoon | Margarine, all types | 112 |
| 1 teaspoon | Olive oil or canola oil (not including olive oil with zaatar) | 113 |
| 1 teaspoon | Other fats | 114 |
| 1 teaspoon | Mayonnaise, all types | 115 |
| 1 teaspoon | Salad dressing | 116 |
| Swipe with 1/2 pita or 1 slice of bread | Olive oil with zaatar | 117 |
| 1 teaspoon | Sauce for cooking | 118 |
|  | Other, specify:___________________________________ | 119 |
|  | Other, specify:___________________________________ | 120 |

# Supplementary Method -4: Urine handling

The total urine volume was recorded, as was time of commencement and completion of 24-hour urine collection. Where urine volume did not exceed 2 liters, the bottle was first mixed and a sample was then drawn, by syringe, from the one bottle of the 24-hour urine collection and placed in a sterile urine collection cup. In cases where urine volume exceeded 2 liters, thereby necessitating use of more than one bottle, a urine sample was drawn from each bottle, in proportion to the amount in each bottle. Samples were then immediately transferred to Tel-Aviv Sourasky Medical Center laboratory for analysis and data entry.

# Supplementary Method -5: Data completion

The rate of missing data was 0.3% for the twenty-four-hour dietary recall questionnaire; 4.9% for the FFQ questionnaire; 5.8% for measured blood pressure; 2.6% for weight and height.

# Supplemental Online Figures

***Figure S1: The survey sampling and recruitment flow***


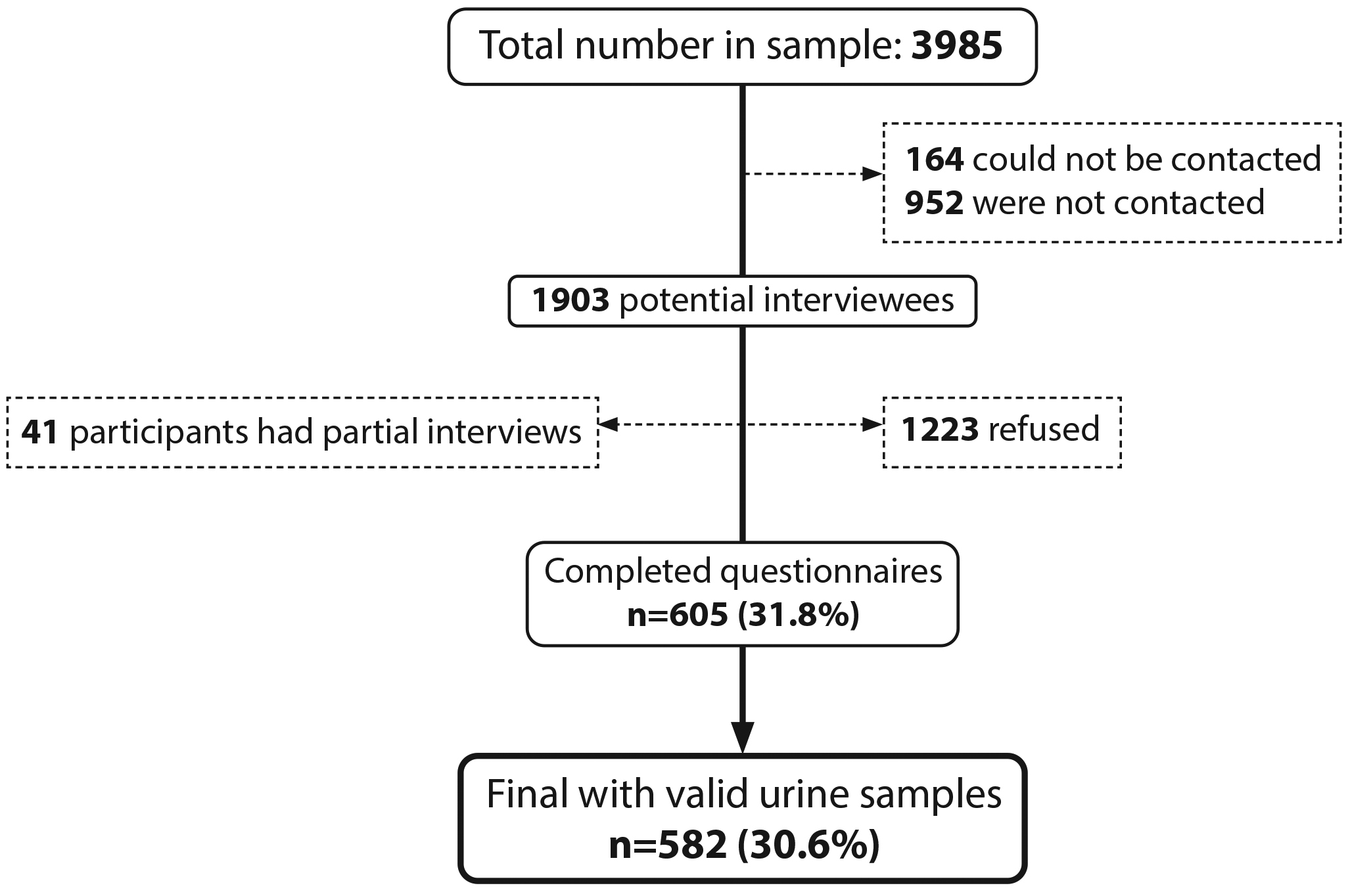


***Figure S2: Contribution (presented as % of total reported sodium intake) of food groups to total sodium intake (As reported in 24-hour recalls)***

***Figure S3: The interaction between energy intake and BMI in relation to sodium excretion – a two-way ANOVA analysis***


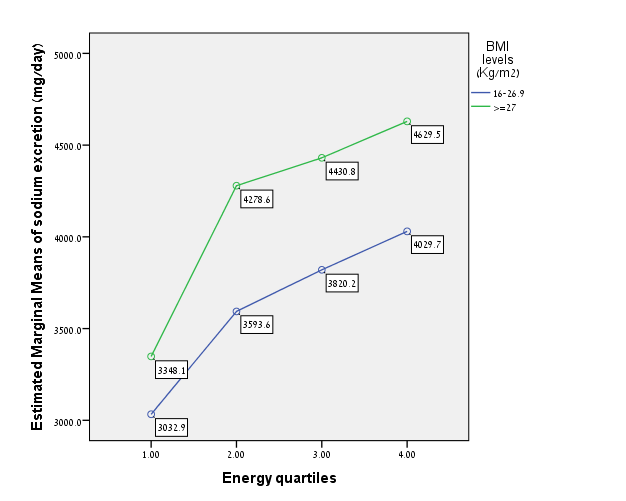


Indicated by the relatively parallel lines, as well as by Between-Subjects Effects Test, there was no overall interaction between caloric intake and BMI in relation to sodium excretion (p=0.802) suggesting that the joint effect of BMI and caloric intake the combined is less than the sum of their individual effects (no synergistic effect on sodium excretion). Error variance of the sodium excretion is equal across groups using the Levene's Test for homogeneity (p=0.053).

***Figure S4: Sodium excretion stratified by measured hypertension and obesity vs non-obesity status***

NS

***P=0.042***

NS
